# Discovery and Analysis of MicroRNAs in *Leymus chinensis* under Saline-Alkali and Drought Stress Using High-Throughput Sequencing

**DOI:** 10.1371/journal.pone.0105417

**Published:** 2014-11-04

**Authors:** Junfeng Zhai, Yuanyuan Dong, Yepeng Sun, Qi Wang, Nan Wang, Fawei Wang, Weican Liu, Xiaowei Li, Huan Chen, Na Yao, Lili Guan, Kai Chen, Xiyan Cui, Meiying Yang, Haiyan Li

**Affiliations:** 1 Ministry of Education Engineering Research Center of Bioreactor and Pharmaceutical Development, Jilin Agricultural University, Changchun, Jilin, China; 2 College of Life Sciences, Jilin Agricultural University, Changchun, Jilin, China; 3 High School attached to Northeast Normal University, Changchun, Jilin, China; Universidade Federal do Rio Grande do Sul, Brazil

## Abstract

*Leymus chinensis* (Trin.) Tzvel. is a perennial rhizome grass of the Poaceae (also called Gramineae) family, which adapts well to drought, saline and alkaline conditions. However, little is known about the stress tolerance of *L. chinensis* at the molecular level. microRNAs (miRNAs) are known to play critical roles in nutrient homeostasis, developmental processes, pathogen responses, and abiotic stress in plants. In this study, we used Solexa sequencing technology to generate high-quality small RNA data from three *L. chinensis* groups: a control group, a saline-alkaline stress group (100 mM NaCl and 200 mM NaHCO_3_), and a drought stress group (20% polyethylene glycol 2000). From these data we identified 132 known miRNAs and 16 novel miRNAs candidates. For these miRNAs we also identified target genes that encode a broad range of proteins that may be correlated with abiotic stress regulation. This is the first study to demonstrate differentially expressed miRNAs in *L. chinensis* under saline-alkali and drought stress. These findings may help explain the saline-alkaline and drought stress responses in *L. chinensis*.

## Introduction

*Leymus chinensis* (Trin.) Tzvel. is a perennial rhizome grass of the Poaceae family, which is distributed widely throughout the northern temperate areas of Eurasia [1]. *L. chinensis* is cultivated as a major grass forage product for its high protein content, productivity, palatability and nourishment. Additionally, because *L. chinensis* adapts well to drought, saline, alkaline and low temperature conditions, it is cultivated particularly on alkaline land, which means it has an important role to play in the protection of the environment [2]. However, the genome sequence of *L. chinensis* has not yet been published, and very little genetic information is publicly available. A few studies have investigated the saline-alkaline tolerance of *L. chinensis* at the molecular level, but no microRNA (miRNA) studies have been reported to date.

miRNAs are non-coding RNAs, approximately 21–26 nucleotides (nt) long, that play crucial roles in the regulation of gene expression in plants [3]. Several plant miRNAs that are involved in stress responses are regulated by abiotic stresses, including drought, high salinity, and low temperatures. For example, the expression levels of rice miR393 change under salinity and alkaline stresses and its putative target genes are related to abiotic stress [4], while miR169 plays an essential role in drought resistance in tomato [5]. Additionally, recent studies have shown that a number of other differentially expressed miRNAs are involved in stress regulation [6], [7], [8]. Overall, these studies suggest that miRNAs and their target mRNAs play important roles in stress tolerance. It is becoming increasingly evident that miRNAs play an important role in plant development and resistance to biotic as well as abiotic stresses.

Various methods have been used to systematically discover miRNAs in rice, wheat and maize [9], [10], [11], [12]. To provide further insights into the role of miRNAs in *L. chinensis* tolerance to stress, we analyzed the expression patterns of miRNAs from *L. chinensis* exposed to drought and saline-alkali stresses using Solexa high-throughput sequencing technology and quantitative real time PCR (qRT-PCR). We identified and analyzed 132 known miRNAs and 16 novel miRNA candidates of *L. chinensis*. Fifteen and 19 miRNAs were differentially expressed under saline-alkali and drought stress, respectively, relative to control. These results indicated that these miRNAs were associated with drought, salinity and alkaline stress. We verified variations in the expression profiles of several miRNAs during saline-alkali and drought stresses using qRT-PCR analysis. We report the differential expression of *L. chinensis* miRNAs that may be involved in saline-alkaline and drought stress regulation. This study has important implications for gene regulation in plants under saline-alkaline and drought stress conditions.

## Materials and Methods

### 1. Sample treatment and RNA isolation

Seeds of *L. chinensis* (Jisheng No.4 Chinese Wildrye), which has high saline-alkaline and drought resistance, were obtained from the Jilin Province Jisheng Wildrye Excellent Seed Station. The seeds were surface sterilized with 75% ethanol and reared in an artificial climate chamber in Hoagland nutrient solution. The nutrient solution was changed every 2 days. When the “3-leaves” stage was reached, the seedlings were transferred either into Hoagland nutrient solution with 100 mM NaCl and 200 mM NaHCO_3_ to simulate salinity and alkaline stresses or into Hoagland nutrient solution containing 20% polyethylene glycol 2000 (PEG-2000) to simulate drought stress. Some plants were cultivated in Hoagland nutrient solution as a control. The seedlings were treated for 24 h, then frozen in liquid nitrogen and stored at −80°C. Total RNA was extracted with Trizol reagent (Invitrogen, CA, Carlsbad, USA) according to the manufacturer's instructions. All the experiments followed a randomized design and were conducted using three replicates of each of the samples.

### 2. Small RNA sequencing library construction

RNAs less than 30 nucleotides long were selected and purified by 15% denaturing polyacrylamide gel electrophoresis (PAGE). A 5' chimeric oligonucleotide adaptor primer (5′-pUCGUAUGCCGUCUUCUGCUUGidT-3′) and a 3′ chimeric oligonucleotide adaptor primer (5′-GUUCAGAGUUCUACAGUCCGACGAUC-3′) were ligated to the small RNAs (sRNAs) using T4 RNA ligase and the resulting ligation products were gel purified by 10% PAGE. cDNA libraries were prepared by reverse transcription from the RNA products. The resulting libraries were sequenced on the Solexa Genome Analyzer II platform (Illumina, San Diego, CA, USA) according to the manufacturer's instructions. All the short reads were deposited in the National Center for Biotechnology Information (NCBI) and can be accessed in the Short Read Archive (SRA) under the accession number, SRP033396.

### 3. Bioinformatic analysis

After Solexa sequencing, low-quality reads were removed and the remaining high-quality sRNA reads were filtered to remove low quality tags and adaptor sequences. We called the filtered set of sequences the clean sequences. To identify known miRNAs, the clean sequences were searched against the non-coding RNAs (rRNA, tRNA, snRNA and snoRNA) in the Rfam 9.1 (http://rfam.janelia.org) and miRBase 19.0 (http://www.mirbase.com) databases using BLASTN with a threshold of at least 95% identity and 90% coverage (E-value ≤1e-5). To identify novel miRNAs, the sRNA reads that had matches in Rfam and miRBase were eliminated and the remaining reads were aligned to the *L. chinensis* mRNA transcript database [13] using SOAP 2.20 software (http://soap.genomics.org.cn). The SOAP2.2 output was filtered with a precursor tool to separate candidate sequences as miRNA precursors with an anchoring pattern of a block of aligned small RNAs with perfect matches. MIREAP software (http://sourceforge.net/projects/mireap/) was then used to identify candidate primary miRNAs on the genome sequence flanking the identified miRNAs. The *L. chinensis* candidate miRNAs that were detected using this approach were named according to miRBase. The RNAfold web server (http://rna.tbi.univie.ac.at/cgi-bin/RNAfold.cgi) was used to evaluate whether the predicted primary miRNAs could form suitable hairpin structures. Folding free energies (MFE) were also used to evaluate these candidate novel miRNAs. The number of reads for each identified miRNA was normalized against the total number of reads in the corresponding library to determine the expression level of the miRNA. Next, a Bayesian method was used to evaluate the statistical significance of expression differences between different samples. *P* values ≤0.01 and changes of at least two-fold were used as the threshold to confirm statistical significance. Candidate targets of the known and novel miRNAs were predicted by psRNATarget using *L. chinensis* assembled unigenes (http://bioinfo3.noble.org/psRNATarget/). The criteria used for psRNATarget were as follows: sequences were considered to be miRNA targets when the total score was less than four mismatches (a G:U wobble pair was assigned a mismatch score of 0.5). BLASTX searches were performed using the mRNA sequences of the predicted miRNA targets to query the NCBI protein database to predict the functions of potential targets. The COG (clusters of orthologous groups) terms for the target genes were assigned by comparing the NCBI sequences that were obtained against the COG database (http://www.ncbi.nlm.nih.gov/COG).

### 4. miRNA validation and expression analysis by qRT-PCR

To validate expression levels of miRNAs, qRT-PCR was performed for the candidate *L. chinensis* miRNAs from the three libraries. The forward miRNA primers were designed based on the full-length miRNA sequences, and the reverse primer was the universal reverse primer for miRNAs [14]. The primers are listed in Table S1. Total RNA (1 µl) in 20 µl reaction volumes was used to synthesize reverse transcripts using a One Step PrimeScript miRNA cDNA Synthesis Kit (Takara, Japan). 5 s rRNA was used as the reference gene. The PCR conditions were as follows: an initial polymerase activation step for 3 min at 94°C, 20 cycles of 15 s at 94°C for denaturation, 20 s at 66°C for annealing, and 60 s at 72°C for elongation.

To validate expression patterns of miRNAs and their targets, qRT-PCR was performed for the predicted unigenes. Primer sequences are given in Table S2. The 25 µl reactions contained 2 µl cDNA, 12.5 µl 2× SYBR premix ExTaq (Takara, Japan), and 10 µM of the forward and reverse primers. The thermal cycling conditions were 40 cycles at 95°C for 5 s for denaturation and 60°C for 20 s for annealing and extension.

The amplification reaction was performed using the ABI7300 real-time PCR system (Applied Biosystems, Foster City, CA, USA). To calculate the relative expression of miRNAs and unigenes, the 2^−ΔΔCt^ value was calculated and transformed into fold-change differences.

### 5. 5'-RACE validation of target genes

To validate the miRNA target genes, rapid amplification of cDNA ends (RACE) assays were performed using a 5′-RACE kit (Takara, Dalian, China) [15], [16]. Total RNA was treated with tobacco acid pyrophosphatase at 37°C for 1 h. A RNA Oligo adapter was then directly ligated to the treated RNAs and two gene-specific primers were used for RACE (Table S3). The PCR products were visualized on agarose gels. Bands were excised and sequenced to determine the 5′ ends of the transcripts.

## Results

### 1. Overview of Solexa sequenced sRNA libraries from *L. chinensis* grown under stress

**Figure 1:**
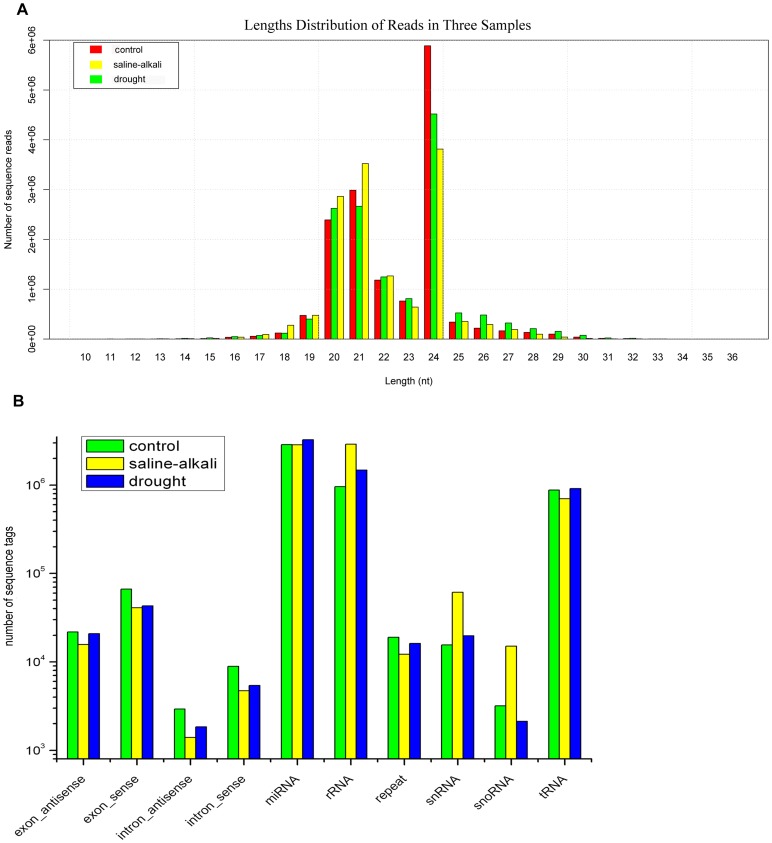
Length distribution and proportions of various categories of small RNAs in *L. chinensis* in the libraries from control, saline-alkaline and drought treated plants. A: Length distribution of reads in the three libraries; B: Categories of small RNAs in *the* three libraries.

Solexa sequencing of the *L. chinensis* libraries generated a large number of short reads (10–40 nt long). The control library yielded 15,712,504 unfiltered raw sequence reads, and the saline-alkali and drought libraries yielded 15,076,385 and 14,725,482 raw reads, respectively. After filtering to remove low-quality reads, the control library contained 15,301,799 high-quality reads, and after filtering out the 3′-adapter, 5′-adapter and poly(A) reads, we obtained 14,825,707 clean sequences, which accounted for 96.89% of the high-quality reads. For the stress-induced libraries, we obtained 14,190,211 clean sequences from the saline-alkali library and 13,856,136 clean sequences from the drought library, which accounted for 96.90% and 96.67% of the high-quality reads in the two libraries, respectively (Table 1). Most of the sequence tags varied in length from 19 nt to 30 nt, (Figure 1A). The overall distribution of the redundant small RNAs are similar in the three samples, with one major peak at 24 nt and another minor peak at 20 nt and 21 nt. While in drought samples, the propotion of 24 nt small RNAs decreased and the 20-nt or 21-nt populations increased to a major peak.

**Table 1:** Read quality in the control and stress-induced libraries.

	Control	saline-alkaline stress group	drought stress group
Raw reads	15,712,504	15,076,385	14,725,482
high quality	15,301,799	14,644,590	14,332,735
3′adapter_null	212,414	266,964	158,349
insert_null	4,465	2,686	6,507
5′adapter_contaminants	148,528	21,684	153,276
smaller_than_18 nt	108,118	162,419	155,359
polyA	2,567	626	3,108
clean_reads	14,825,707	14,190,211	13,856,136

In the control library, the 24 nt sRNAs were the most abundant, making up 39.42% of the clean sequences; in the saline-alkali and drought libraries, the 24 nt sRNAs made up 27.22% and 31.48% of the clean sequences, respectively. The 20 nt and 21 nt sRNAs were also abundant, making up 36.02%, 45.58% and 36.85% of the clean sequences in the control, saline-alkali and drought libraries respectively. It is worth noting that 24 nt, 20 nt and 21 nt are typical lengths for miRNAs (16–29 nt). Some 22 nt sRNAs were also present in the control, saline-alkali and drought libraries; however, they made up only 7.92%, 9.05% and 8.69% of the clean sequences, respectively.

There are several kinds of sRNA, including snRNAs, snoRNAs, rRNAs, tRNAs, and miRNAs (Figure S1). When all the clean sequences were searched against the GeneBank, Rfam, and miRbase databases, we found that a relatively small proportion of them mapped to known sequences. The unmatched sequences (53–67%) were characterized as unclassified sRNAs. A proportion of the clean sequences, 36.66%, 46.48% and 41.52% from the control, saline-alkali and drought libraries respectively, partially mapped to known non-coding RNA sequences (Figure 1B) and about 20% of the mapped sequences from each of the libraries were identified as miRNAs. These numbers imply that there was a relatively high abundance of miRNAs in the *L. chinensis* libraries; the unclassified sRNAs may represent either new miRNAs or other classes of non-coding RNAs.

### 2. Identification of conserved miRNAs expressed under saline–alkaline and drought stress

We used the clean sequences to search the miRBase 19.0 database to identify mature miRNAs, miRNA stars (a miRNA star, miRNA*, denotes an sRNA processed from the hairpin arm opposite the mature miRNA), and precursor miRNAs in the *L. chinensis* libraries. Because known miRNA sequences of *L. chinensis* are scarce, the miRNAs and pre-miRNAs of rice (*Oryza sativa* Linn.) and barley (*Hordeum vulgare*) were used as the reference sequences against which the miRNAs from *L. chinensis* were mapped. We identified 132 unique known miRNAs in the three libraries, which were distributed in 38 conserved miRNA families (Table S4). The read counts for the sequences in each of the libraries that mapped to the known miRNAs (reads number shown in figure 1B) suggested that the sequencing reads were sufficient to support an estimation of the expression levels of the identified miRNAs in the three libraries.

We found some notable quantitative differences for the known miRNAs in the stress-induced libraries. The abundance of the individual miRNAs was calculated relative to the total number of identified miRNA reads in the corresponding library. We detected 13 miRNAs for which the read counts were more than 400,000 in all three libraries. The most abundant miRNAs across the three libraries were members of the miR156 family, including lch-miR156d, lch-miR156g, lch-miR156h and lch-miR156b. Of these, the most abundant was miR156d with 2,039,447 clean sequences in the control library, followed by lch-miR156g, lch-miR156h and lch-miR156b, all with more than 200,000 read counts each. lch-miR156d is one of the most conserved miRNAs having been identified in 12 different species (*Arabidopsis thaliana*, *Selaginella moellendorffii, Zea mays, Sorghum bicolor, O. sativa, Vitis vinifera, Populus trichocarpa, Gossypium hirsutum, Medicago truncatula, Glycine max, Ricinus communis* and *Arabidopsis lyrata*). Similar observations were made for the members of three other miRNA families, miR168, miR166 and miR167. Each family had more than 20,000 clean sequences in each of the three libraries; among them, lch-miR168-5p and lch-miR168a, with more than 400,000 sequences each, were the most abundant. lch-miR168-5p was conserved in *Phaseolus vulgaris* and lch-miR168a was conserved in *Z. mays, S. officinarum, O. sativa, P. trichocarpa, A. lyrata, A. thaliana* and *S. bicolor*. These two high abundance miRNAs were present in similar abundances in all three libraries. The majority of the high abundance miRNAs were differentially expressed in the different libraries. For example, miR166e with more than 56,000 clean sequences was expressed at a high level, while lch-miR166b, lch-miR166c and lch-miR169n, which also showed high expression levels, were expressed in all three libraries. Similarly, lch-miR399a, a highly expressed miRNA, was found in all three libraries. The different abundances of the different members of a miRNA family may suggest that they have varied regulatory roles under the different environmental conditions.

### 3. Identification of novel miRNA candidates

Because the genome of *L. chinensis* has not yet been sequenced, the identification of novel miRNAs can be a challenge. We used methods previously described to construct a non-redundant database of *L. chinensis* novel miRNA candidates by selecting the most abundant sequence as the representative for each set of similar sequences.

Sixteen novel miRNAs from the three libraries were identified as miRNA candidates (Table 2). These novel miRNAs length ranged from 20 to 23 nt and the lower minimal folding free energy (MFE) ranged from −19.1 to −110.7. These candidate sequences were among the most highly expressed sRNAs in the *L. chinensis* libraries, indicating that they are likely to play a major functional role in the plants' responses to stress. Based on their abundances and sequences, we found that these novel miRNAs displayed lower expression levels than the majority of miRNAs belonging to known families. The low abundance of the novel miRNAs might suggest specific roles for these miRNAs under the stress conditions.

**Table 2:** Novel miRNAs predicted from the three *L. chinensis* libraries.

Novel miRNA	miRNA sequence	3p/5p	strand (±)	length (nt)	folding energy (kcal/mol)
lch-MIR-01	TCTTATATTATTGGACGGAGG	3p	+	21	−72.5
lch-MIR-02	AAGTAATTTGAGACGGAGGGAGT	3p	+	23	−58.35
lch-MIR-03	AAAACATCAACAATCGGAACTTA	5p	-	23	−31.03
lch-MIR-04	GTGTGGCGGCATGGGGATGT	3p	+	20	−42.1
lch-MIR-05	TGAGAGTGCGAATACAAGGAGGT	5p	-	23	−21.7
lch-MIR-06	ATTTCTGGACGGAGGGAGTAT	5p	+	21	−19.1
lch-MIR-07	AAGAGTAGCGTTGATACACCGT	5p	-	22	−68
lch-MIR-08	AAGAATTATGGAATGGAGGGA	3p	-	21	−56.3
lch-MIR-09	AAGACAAGTAATTTGGGACGG	3p	+	21	−45.5
lch-MIR-10	CTCTTATTGAATCGCGGTAAAGT	3p	-	23	−58.3
lch-MIR-11	ATGTGTGTGTCTGTGTGTGTA	5p	-	21	−110.7
lch-MIR-12	TTCTTCGACTTGGCCATCTCCC	5p	+	21	−61.3
lch-MIR-13	AATAGGTAGGGATGACAGGATT	5p	+	22	−42.5
lch-MIR-14	GGGACGAGTTGGAAGAGGAAT	3p	+	21	−22.5
lch-MIR-15	AGGCTGTGGAGAGATGGCTGAGT	3p	-	23	−26.4
lch-MIR-16	TTTAGGAACGGAGGAAGTACA	5p	-	21	−52.6

### 4. Different expression patterns of miRNAs under saline–alkaline and drought stress conditions

miRNAs make up a large majority of the *L. chinensis* small RNA libraries when compared with other kinds of sRNA. Some identified miRNAs had more than one million sequencing reads, while others had fewer than one hundred. The most abundant miRNA was miR156. miR156d had 2,039,447 reads in the control library and 2,147,423 and 2,347,874 reads in the saline-alkaline and drought libraries, respectively. This high level of expression correlates with evolutionary conservation [17]. In contrast, some conserved miRNAs, such as miR156l, were found to be expressed at low levels compared with miR156d. It is likely that theses miRNAs have specific expression patterns in *L. chinensis* under stress conditions.

**Figure 2:**
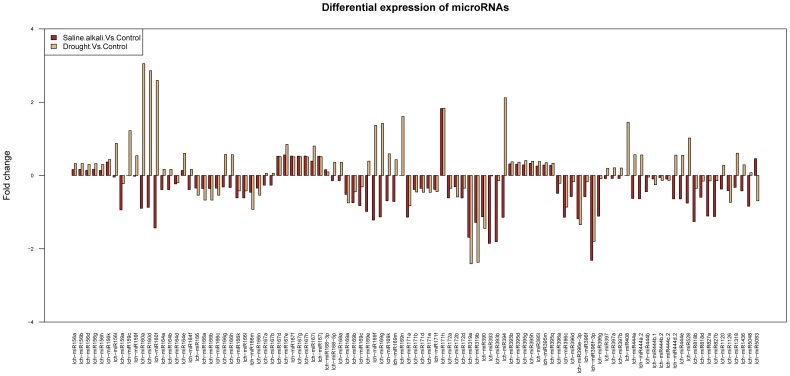
Differentially expressed miRNAs in the stress-induced libraries compared with the control.

To compare differential expression patterns between control and stressed libraries, standardized treatments of miRNA abundance were performed. The common expression patterns of *L. chinensis* miRNAs under saline-alkaline and drought stress conditions are shown in Figure 2. miRNA expression varied in response to different stress-inducing conditions. Fifteen miRNAs (lch-miR160f, lch-miR171a, lch-miR171h, lch-miR319a, lch-miR319b, lch-miR390, lch-miR393, lch-miR393b, lch-miR394, lch-miR396c, lch-miR396e-3p, lch-miR396f-3p, lch-miR396g, lch-miR827a, lch-miR827b) were identified as differentially expressed with fold-changes >2 between control and saline-alkaline libraries (*p* value <0.1). Fourteen of them were significantly differentially expressed (*p* value <0.01) (lch-miR394 was not). Nineteen miRNAs were differentially expressed under drought stress with fold-changes >2 when compared with the control library (*p* value <0.05); seven of these (lch-miR160a, lch-miR160d, lch-miR160f, lch-miR319a, lch-miR319b, lch-miR394 and lch-miR396f-3p) were significantly differentially expressed (*p* value <0.01). lch-miR160a differed by a >8 fold-change between control and drought. The expression profiles of only a minority of the differentially expressed miRNAs were expressed at the same level, suggesting that differential miRNA expression might partly contribute to stress regulation among the various treatments.

### 5. Validation of the miRNA expression profiles by qRT-PCR

**Figure 3:**
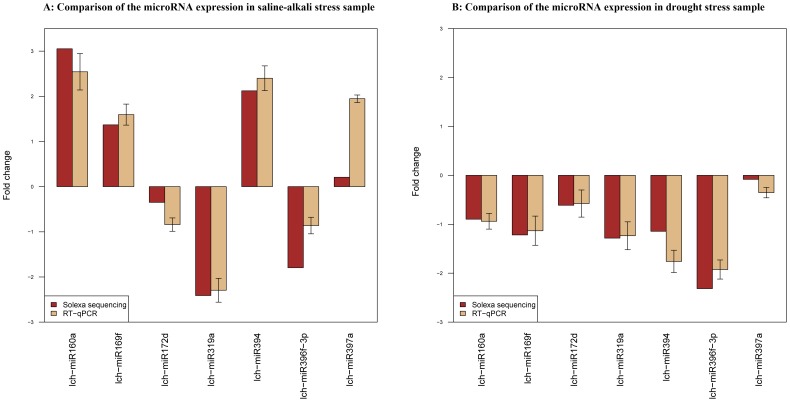
Comparison of the miRNA expression levels determined by Solexa sequencing and qRT-PCR. A: Comparison of miRNA expression in the saline-alkali stress sample; B: Comparison of miRNA expression in the drought stress sample.

Stem-loop qRT-PCR was used to measure and validate the expression levels of seven randomly selected *L. chinensis* miRNAs (lch-miR160a, lch-miR169f, lch-miR172d, lch-miR319a, lch-miR394, lch-miR396f-3p and lch-miR397a) in the three libraries. The expression levels of these miRNAs measured by qRT-PCR were compared with those obtained by Solexa analyses of the libraries and the two sets of results were consistent (Figure 3).

### 6. Predicted targets of the known and novel miRNAs

Understanding the biological roles of the *L. chinensis* miRNAs requires the identification of their mRNA targets [18], [19]. We used psRNATarget to predict the mRNA targets of the conserved and novel (Table S5, Table S6) miRNAs using assembled transcripts of *L. chinensis*. We used 3.5 as a cut off threshold and found 125 putative targets for 24 conserved miRNA families and 59 targets for 12 novel miRNAs.

**Figure 4:**
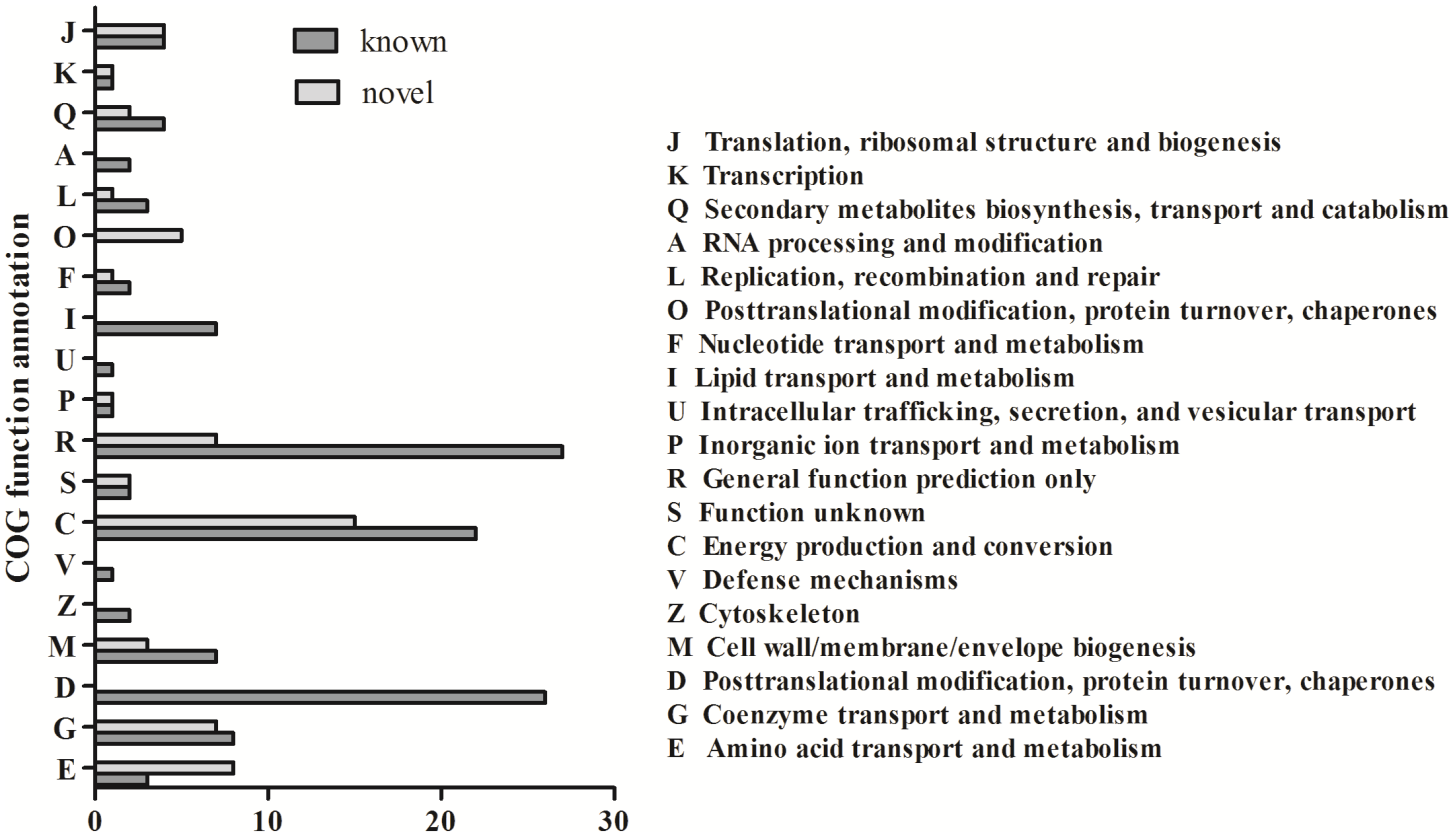
COG functional classification of the miRNA target genes identified in the *L. chinensis* libraries.

The functions of all putative targets were identified by COG analysis (figure 4). Some of the predicted targets were coproporphyrinogen III oxidase, glutathione S-transferase, ubiquitin-protein ligase, MYB superfamily members, and several NAD-dependent aldehyde dehydrogenases, indicating that these predicted target genes encode a broad range of proteins correlated with metabolic and cellular processes. We also found eight putative targets for conserved miRNAs and one putative target for one of the novel miRNAs involved in abiotic stress regulation. These target genes may be involved with oxidative stress, which can damage cellular structures, in response to water deficit and pathogen attack [20], [21].

**Figure 5:**
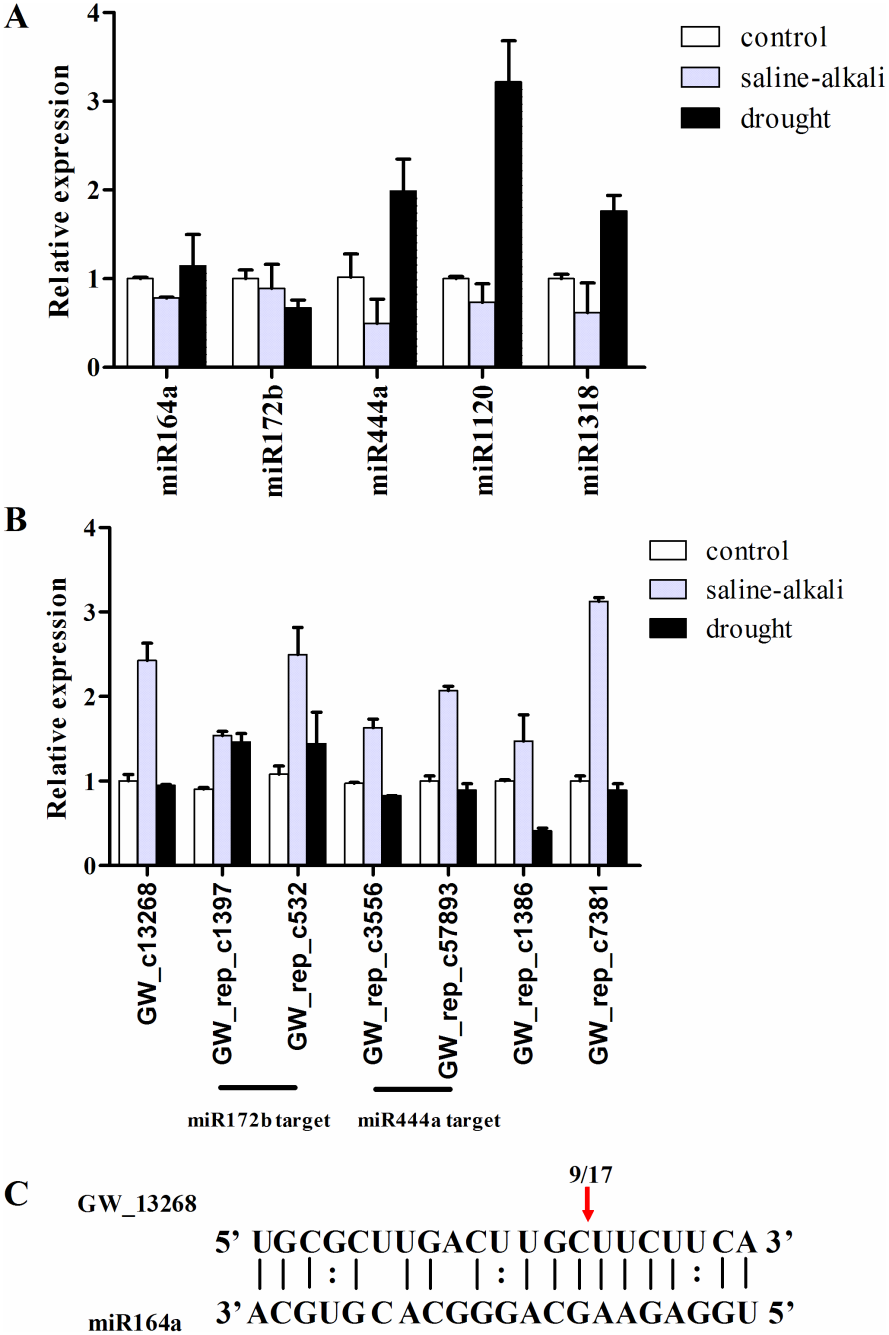
Analysis of potential targets in *L. chinensis*. A: Characterization of candidate miRNA expression levels under saline-alkali and drought stress by qRT-PCR; B: Comparison of predicted target expression levels under saline-alkali and drought stress by qRT-PCR; C: Cleavage site analysis of GW_13268 by 5′ RACE PCR.

We analyzed the expression of the predicted targets of known *L. chinensis* miRNAs using published transcriptome sequencing data [13]. Under saline-alkaline stress, we observed no change in the expression for four predicted targets, GW_c49847, GW_c34555, GW_rep_c87771 and GW_c41075, while 109 predicted targets were up- or down-regulated. Among them, 68 had similar expression patterns to the miRNAs that regulate them (table S4, table S7). We then analyzed five miRNAs (miR164a, miR172b, miR444a, miR1120 and miR1318) and their predicted targets (from the 68 described above) by qRT-PCR. After considering the miRNA expression patterns, seven target genes showed a moderate increase in expression under both saline–alkaline and drought conditions (Figure 5A, 5B). Finally, analysis of GW_c13268 by 5′-RACE revealed an mRNA fragment consistent with miRNA-guided cleavage in agreement with previous results obtained for predicted targets of known miRNAs (Figure 5C).

## Discussion

Many recent studies have demonstrated that plant miRNAs are involved in environmental stress tolerance [22] and some stress-regulated miRNAs have been shown to be involved in the cell response to abiotic stresses such as salinity, cold and dehydration [23], [24]. Much attention has been devoted to the identification of components involved in signal transduction pathways, such as abscisic acid signaling, because they are known to participate in responses against the adverse effects of different stresses, such as salinity and drought [25], [26]. Stress response-specific miRNAs and some stress-responsive target genes were observed in previous studies [27], [28]; for example, in rice, osa-miR169g was reported to be the only miRNA member of the miR169 family induced by drought [23].

Application of the newly developed high-throughput sequencing technologies has led to the identification of entire sets of miRNAs, which has delivered new insights into the role of miRNAs in plant development, and stress-related regulation. However, until now, little was known about the functions of miRNAs in abiotic stress responses in *L. chinensis*. Here, we identified 132 known miRNAs and 16 novel miRNAs after sequencing and analyzing the sRNAs of *L. chinensis*. Many miRNAs with a wide range of expression levels were found in the control, drought and saline-alkaline libraries. The most abundantly expressed miRNA family across the three libraries was miR156, which includes miR156d, miR156f, miR156h, miR156b (Table S2). miR156d, miR156f and miR156h are conserved in many species and they exhibited high expression levels in all three *L. chinensis* libraries. The most abundant was miR156d with 2,039,447 reads in the control library. Some miRNAs were differentially expressed between the two stress-induced libraries (Figure 2) and nine of them were significantly differentially expressed under the two stress conditions. Under the saline-alkaline stress conditions, four (lch-miR160a, lch-miR160d, lch-miR160f and lch-miR394) were significantly up-regulated with fold-changes >4 and two (lch-miR319a and lch-miR319b) were significantly down-regulated with fold-changes >4. lch-miR396f-3p was significantly down-regulated under the drought stress conditions. Previous reports found that members of the miR319 and miR393 families were stress inducible [29], [30], [31]. Therefore, we speculate that miR160, miR319a and miR396f-3p can be involved in a multi-stress response to saline-alkali conditions based on our results, and also to conditions that induce maintenance of the energy supply based on other reports [11], [22]. The qRT-PCR results confirmed the up- and down-regulated trends of the differentially expressed miRNAs in the stress-induced libraries (Figure 3); in particular, lch-miR319a, lch-miR396f-3p and lch-miR397a were predicted to be associated with saline-alkaline and drought stresses.

Because the differential expression of the known miRNAs was less significant, we focused our analyses on the novel miRNAs. Deep sequencing of sRNA transcriptomes yields an incredible amount of data, which can be used to characterize not only known miRNAs, but also novel miRNAs with high accuracy and efficiency. Because the *L. chinensis* genome has not yet been sequenced, we used complex network methods to construct a non-redundant database of miRNA candidates by selecting the most abundant sequences and aligning them with the rice genome sequence. From the three *L. chinensis* sRNA libraries, 16 miRNAs were revealed as candidate novel miRNAs. The most highly expressed sRNAs in *L. chinensis* are likely to have important functional roles in the plant's stress response.

In plants, the response to abiotic stresses is complex and involves many biochemical and molecular mechanisms that are regulated mainly through the silencing or regulation of target genes by miRNAs. miRNAs as regulators of target genes have been reported to play major roles in a plant's response to abiotic stresses, including dehydration, freezing, salinity, alkalinity [32], [33]. The identification of miRNA targets is crucial for understanding the biological effects of miRNAs. The identification of the entire set of miRNAs and their targets from an organism is of fundamental importance for understanding overall gene regulation involving stress responses.

In this study, target genes for miRNAs that were differentially expressed in the three libraries were identified by a search of plant miRNA target sites, which are predominantly located in open reading frames. The predicted miRNA target genes included coproporphyrinogen III oxidase, glutathione S-transferase, ubiquitin-protein ligase, MYB superfamily members, NAD-dependent aldehyde dehydrogenases and genes involved in RNA processing. In *L. chinensis*, these target genes may participate in various aspects of plant development and stress responses. miR159 [34], [35], miR160 [36], [37], miR167 [38], miR319 [30], [39], miR393 [31], [40], [41], [42], [43] and miR408 [44], [45], [46] participate in important and perhaps conserved functions, such as plant growth, development and stress responses. MYB transcription factors regulated by miR159 are involved in the biosynthesis of bioactive compounds, such as ABA [47]. In this study we identified miR159f, which may target components (ribose 5-phosphate isomerase, FAD/FMN-containing dehydrogenases) that were down-regulated under saline-alkali stress. Together with previous evidence for the targeting of MYB by miR159, these predicted targets may be up-regulated and regulated by the ABA signaling pathway. The results suggest that miRNAs may regulate ABA signaling and other stress response processes. Two miR319 members (miR319a and miR319b) were down-regulated under both saline-alkali and drought stress. miR319 was reported as a positive regulator of cold stress in rice by targeting two genes (PCF6 and TCP 21) [30]; however, these targets were not predicted from our *L. chinensis* transcript results. We predicted another two targets of miR319 (GW_rep_c24088/GW_rep_c74568, GW_rep_c58089). miR319 promotes cleavage of GW_rep_c58089 despite having a complementary site within the ORF of its target. Further qRT-PCR analysis revealed that GW_rep_c58089 is up-regulated under drought stress. Because of opposite expression patterns of miR319b and GW_rep_c58089, we suggest that GW_rep_c58089 annotated as ribulose bisphosphate carboxylase small subunit may be another target regulated in saline-alkali and drought stress in *L. chinensis*. Over-expression of miR393 in rice leads to less tolerance to salt and suggests that salt stress may cause decreased TIR1 and AFB2 mRNA degradation or translational repression [48]. miR393 is down-regulated under saline-alkali stress in our dataset. The identification of miR393, which we predict to target five unknown proteins (Table S3), will be valuable for investigating the function of miRNAs. Further evidence is needed to uncover miRNA functions in the saline-alkali stress response. Although qRT-PCR tested the expression pattern of several miRNAs and their corresponding targets, 5′-RACE PCR identified only one target of miR164a; the other six putative targets were not detected, likely reflecting low abundance and coverage of *L. chinensis* transcripts. The global identification of miRNA target genes will provide useful information to explore the functions of miRNAs in plant biogenesis

## Supporting Information

Figure S1 **Read abundance of various classifications of small RNAs in the** ***L. chinensis*** **libraries.** A: Read abundance of various classifications of small RNAs in the control sample, B: Read abundance of various classifications of small RNAs in the saline-alkali stress sample C: Read abundance of various classifications of small RNAs in the drought stress sample.
(TIF)

Table S1 **microRNA primers for qRT-PCR.**
(DOC)

Table S2 **microRNA and unigene primers for qRT-PCR.**
(DOC)

Table S3 **5′ RACE PCR GSP primer.**
(DOC)

Table S4 **Known miRNAs in the three small RNA libraries.**
(DOC)

Table S5 **Target genes of known miRNAs.**
(DOCX)

Table S6 **Target genes of novel miRNAs.**
(DOCX)

Table S7 **Expression profile of target unigenes.**
(DOC)

## References

[1] Huang Z, Zhu J, Mu X, Lin J (2004) Pollen dispersion, pollen viability and pistil receptivity in Leymus chinensis. Ann Bot 93: 295–301. 14744707 10.1093/aob/mch044 PMC4242205

[2] Shu QY, Liu GS, Xu SX, Li XF, Li HJ (2005) Genetic transformation of Leymus chinensis with the PAT gene through microprojectile bombardment to improve resistance to the herbicide Basta. Plant Cell Rep 24: 36–44. 15657763 10.1007/s00299-004-0908-6

[3] Mallory AC, Vaucheret H (2006) Functions of microRNAs and related small RNAs in plants. Nat Genet 38 SupplS31–36. 16736022 10.1038/ng1791

[4] Gao P, Bai X, Yang L, Lv D, Pan X, et al (2011) osa-MIR393: a salinity- and alkaline stress-related microRNA gene. Mol Biol Rep 38: 237–242. 20336383 10.1007/s11033-010-0100-8

[5] Zhang X, Zou Z, Gong P, Zhang J, Ziaf K, et al (2011) Over-expression of microRNA169 confers enhanced drought tolerance to tomato. Biotechnol Lett 33: 403–409. 20960221 10.1007/s10529-010-0436-0

[6] Jeong DH, German MA, Rymarquis LA, Thatcher SR, Green PJ (2010) Abiotic stress-associated miRNAs: detection and functional analysis. Methods Mol Biol 592: 203–230. 19802598 10.1007/978-1-60327-005-2_14

[7] Shen J, Xie K, Xiong L (2010) Global expression profiling of rice microRNAs by one-tube stem-loop reverse transcription quantitative PCR revealed important roles of microRNAs in abiotic stress responses. Mol Genet Genomics 284: 477–488. 20941508 10.1007/s00438-010-0581-0

[8] Kantar M, Lucas SJ, Budak H (2011) miRNA expression patterns of Triticum dicoccoides in response to shock drought stress. Planta 233: 471–484. 21069383 10.1007/s00425-010-1309-4

[9] Jin W, Li N, Zhang B, Wu F, Li W, et al (2008) Identification and verification of microRNA in wheat (Triticum aestivum). J Plant Res 121: 351–355. 18357413 10.1007/s10265-007-0139-3

[10] Zhu QH, Spriggs A, Matthew L, Fan L, Kennedy G, et al (2008) A diverse set of microRNAs and microRNA-like small RNAs in developing rice grains. Genome Res 18: 1456–1465. 18687877 10.1101/gr.075572.107 PMC2527712

[11] Ding D, Zhang L, Wang H, Liu Z, Zhang Z, et al (2009) Differential expression of miRNAs in response to salt stress in maize roots. Ann Bot 103: 29–38. 18952624 10.1093/aob/mcn205 PMC2707283

[12] McCormick KP, Willmann MR, Meyers BC (2011) Experimental design, preprocessing, normalization and differential expression analysis of small RNA sequencing experiments. Silence 2: 2. 21356093 10.1186/1758-907X-2-2 PMC3055805

[13] Sun Y, Wang F, Wang N, Dong Y, Liu Q, et al (2013) Transcriptome exploration in Leymus chinensis under saline-alkaline treatment using 454 pyrosequencing. PLoS One 8: e53632. 23365637 10.1371/journal.pone.0053632 PMC3554714

[14] Chen C, Ridzon DA, Broomer AJ, Zhou Z, Lee DH, et al (2005) Real-time quantification of microRNAs by stem-loop RT-PCR. Nucleic Acids Res 33: e179. 16314309 10.1093/nar/gni178 PMC1292995

[15] Song C, Wang C, Zhang C, Korir NK, Yu H, et al (2010) Deep sequencing discovery of novel and conserved microRNAs in trifoliate orange (Citrus trifoliata). BMC Genomics 11: 431. 20626894 10.1186/1471-2164-11-431 PMC2996959

[16] Zhu J, Li W, Yang W, Qi L, Han S (2013) Identification of microRNAs in Caragana intermedia by high-throughput sequencing and expression analysis of 12 microRNAs and their targets under salt stress. Plant Cell Rep 32: 1339–1349. 23649877 10.1007/s00299-013-1446-x

[17] Lagos-Quintana M, Rauhut R, Lendeckel W, Tuschl T (2001) Identification of novel genes coding for small expressed RNAs. Science 294: 853–858. 11679670 10.1126/science.1064921

[18] Carlsbecker A, Lee JY, Roberts CJ, Dettmer J, Lehesranta S, et al (2010) Cell signalling by microRNA165/6 directs gene dose-dependent root cell fate. Nature 465: 316–321. 20410882 10.1038/nature08977 PMC2967782

[19] Matts J, Jagadeeswaran G, Roe BA, Sunkar R (2010) Identification of microRNAs and their targets in switchgrass, a model biofuel plant species. J Plant Physiol 167: 896–904. 20207044 10.1016/j.jplph.2010.02.001

[20] Griffiths-Jones S, Grocock RJ, van Dongen S, Bateman A, Enright AJ (2006) miRBase: microRNA sequences, targets and gene nomenclature. Nucleic Acids Res 34: D140–144. 16381832 10.1093/nar/gkj112 PMC1347474

[21] Griffiths-Jones S, Saini HK, van Dongen S, Enright AJ (2008) miRBase: tools for microRNA genomics. Nucleic Acids Res 36: D154–158. 17991681 10.1093/nar/gkm952 PMC2238936

[22] Jones-Rhoades MW, Bartel DP (2004) Computational identification of plant microRNAs and their targets, including a stress-induced miRNA. Mol Cell 14: 787–799. 15200956 10.1016/j.molcel.2004.05.027

[23] Zhao B, Liang R, Ge L, Li W, Xiao H, et al (2007) Identification of drought-induced microRNAs in rice. Biochem Biophys Res Commun 354: 585–590. 17254555 10.1016/j.bbrc.2007.01.022

[24] Sunkar R, Zhou X, Zheng Y, Zhang W, Zhu JK (2008) Identification of novel and candidate miRNAs in rice by high throughput sequencing. BMC Plant Biol 8: 25. 18312648 10.1186/1471-2229-8-25 PMC2292181

[25] Zhu JK (2002) Salt and drought stress signal transduction in plants. Annu Rev Plant Biol 53: 247–273. 12221975 10.1146/annurev.arplant.53.091401.143329 PMC3128348

[26] Seki M, Umezawa T, Urano K, Shinozaki K (2007) Regulatory metabolic networks in drought stress responses. Curr Opin Plant Biol 10: 296–302. 17468040 10.1016/j.pbi.2007.04.014

[27] Prashanth SR, Sadhasivam V, Parida A (2008) Over expression of cytosolic copper/zinc superoxide dismutase from a mangrove plant Avicennia marina in indica rice var Pusa Basmati-1 confers abiotic stress tolerance. Transgenic Res 17: 281–291. 17541718 10.1007/s11248-007-9099-6

[28] Xu DQ, Huang J, Guo SQ, Yang X, Bao YM, et al (2008) Overexpression of a TFIIIA-type zinc finger protein gene ZFP252 enhances drought and salt tolerance in rice (Oryza sativa L.). FEBS Lett 582: 1037–1043. 18325341 10.1016/j.febslet.2008.02.052

[29] Sunkar R, Chinnusamy V, Zhu J, Zhu JK (2007) Small RNAs as big players in plant abiotic stress responses and nutrient deprivation. Trends Plant Sci 12: 301–309. 17573231 10.1016/j.tplants.2007.05.001

[30] Wang ST, Sun XL, Hoshino Y, Yu Y, Jia B, et al (2014) MicroRNA319 positively regulates cold tolerance by targeting OsPCF6 and OsTCP21 in rice (Oryza sativa L.). PLoS One 9: e91357. 24667308 10.1371/journal.pone.0091357 PMC3965387

[31] Gupta OP, Meena NL, Sharma I, Sharma P (2014) Differential regulation of microRNAs in response to osmotic, salt and cold stresses in wheat. Mol Biol Rep 41: 4623–4629. 24682922 10.1007/s11033-014-3333-0

[32] Li H, Dong Y, Yin H, Wang N, Yang J, et al (2011) Characterization of the stress associated microRNAs in Glycine max by deep sequencing. BMC Plant Biol 11: 170. 22112171 10.1186/1471-2229-11-170 PMC3267681

[33] Song QX, Liu YF, Hu XY, Zhang WK, Ma B, et al (2011) Identification of miRNAs and their target genes in developing soybean seeds by deep sequencing. BMC Plant Biol 11: 5. 21219599 10.1186/1471-2229-11-5 PMC3023735

[34] Zheng B, Chen X, McCormick S (2011) The anaphase-promoting complex is a dual integrator that regulates both MicroRNA-mediated transcriptional regulation of cyclin B1 and degradation of Cyclin B1 during Arabidopsis male gametophyte development. Plant Cell 23: 1033–1046. 21441434 10.1105/tpc.111.083980 PMC3082252

[35] Reyes JL, Chua NH (2007) ABA induction of miR159 controls transcript levels of two MYB factors during Arabidopsis seed germination. Plant J 49: 592–606. 17217461 10.1111/j.1365-313X.2006.02980.x

[36] Subramanian S, Fu Y, Sunkar R, Barbazuk WB, Zhu JK, et al (2008) Novel and nodulation-regulated microRNAs in soybean roots. BMC Genomics 9: 160. 18402695 10.1186/1471-2164-9-160 PMC2335117

[37] Bazzini AA, Hopp HE, Beachy RN, Asurmendi S (2007) Infection and coaccumulation of tobacco mosaic virus proteins alter microRNA levels, correlating with symptom and plant development. Proc Natl Acad Sci U S A 104: 12157–12162. 17615233 10.1073/pnas.0705114104 PMC1924585

[38] Liu N, Wu S, Van Houten J, Wang Y, Ding B, et al (2014) Down-regulation of AUXIN RESPONSE FACTORS 6 and 8 by microRNA 167 leads to floral development defects and female sterility in tomato. J Exp Bot 65: 2507–2520. 24723401 10.1093/jxb/eru141 PMC4036516

[39] Spanudakis E, Jackson S (2014) The role of microRNAs in the control of flowering time. J Exp Bot 65: 365–380. 24474808 10.1093/jxb/ert453

[40] Windels D, Bielewicz D, Ebneter M, Jarmolowski A, Szweykowska-Kulinska Z, et al (2014) miR393 is required for production of proper auxin signalling outputs. PLoS One 9: e95972. 24763336 10.1371/journal.pone.0095972 PMC3999107

[41] Mao G, Turner M, Yu O, Subramanian S (2013) miR393 and miR164 influence indeterminate but not determinate nodule development. Plant Signal Behav 8 : doi: 10 4161/psb 26753. 10.4161/psb.26753 PMC4091107 24494229

[42] Chen ZH, Bao ML, Sun YZ, Yang YJ, Xu XH, et al (2011) Regulation of auxin response by miR393-targeted transport inhibitor response protein 1 is involved in normal development in Arabidopsis. Plant Mol Biol 77: 619–629. 22042293 10.1007/s11103-011-9838-1

[43] Si-Ammour A, Windels D, Arn-Bouldoires E, Kutter C, Ailhas J, et al (2011) miR393 and secondary siRNAs regulate expression of the TIR1/AFB2 auxin receptor clade and auxin-related development of Arabidopsis leaves. Plant Physiol 157: 683–691. 21828251 10.1104/pp.111.180083 PMC3192580

[44] Feng H, Zhang Q, Wang Q, Wang X, Liu J, et al (2013) Target of tae-miR408, a chemocyanin-like protein gene (TaCLP1), plays positive roles in wheat response to high-salinity, heavy cupric stress and stripe rust. Plant Mol Biol 83: 433–443. 23864359 10.1007/s11103-013-0101-9

[45] Mutum RD, Balyan SC, Kansal S, Agarwal P, Kumar S, et al (2013) Evolution of variety-specific regulatory schema for expression of osa-miR408 in indica rice varieties under drought stress. FEBS J 280: 1717–1730. 23399101 10.1111/febs.12186

[46] Zhang H, Li L (2013) SQUAMOSA promoter binding protein-like7 regulated microRNA408 is required for vegetative development in Arabidopsis. Plant J 74: 98–109. 23289771 10.1111/tpj.12107

[47] Li C, Lu S (2014) Genome-wide characterization and comparative analysis of R2R3-MYB transcription factors shows the complexity of MYB-associated regulatory networks in Salvia miltiorrhiza. BMC Genomics 15: 277. 24725266 10.1186/1471-2164-15-277 PMC4023596

[48] Xia K, Wang R, Ou X, Fang Z, Tian C, et al (2012) OsTIR1 and OsAFB2 downregulation via OsmiR393 overexpression leads to more tillers, early flowering and less tolerance to salt and drought in rice. PLoS One 7: e30039. 22253868 10.1371/journal.pone.0030039 PMC3254625

