# Supplementary figures and images for: Discovery and Analysis of MicroRNAs in Leymus chinensis under Saline-Alkali and Drought Stress Using High-Throughput Sequencing
Source: PLoS One. 2014 Nov 4;9(11):e105417. doi: 10.1371/journal.pone.0105417 (PMC4219666; doi:10.1371/journal.pone.0105417)

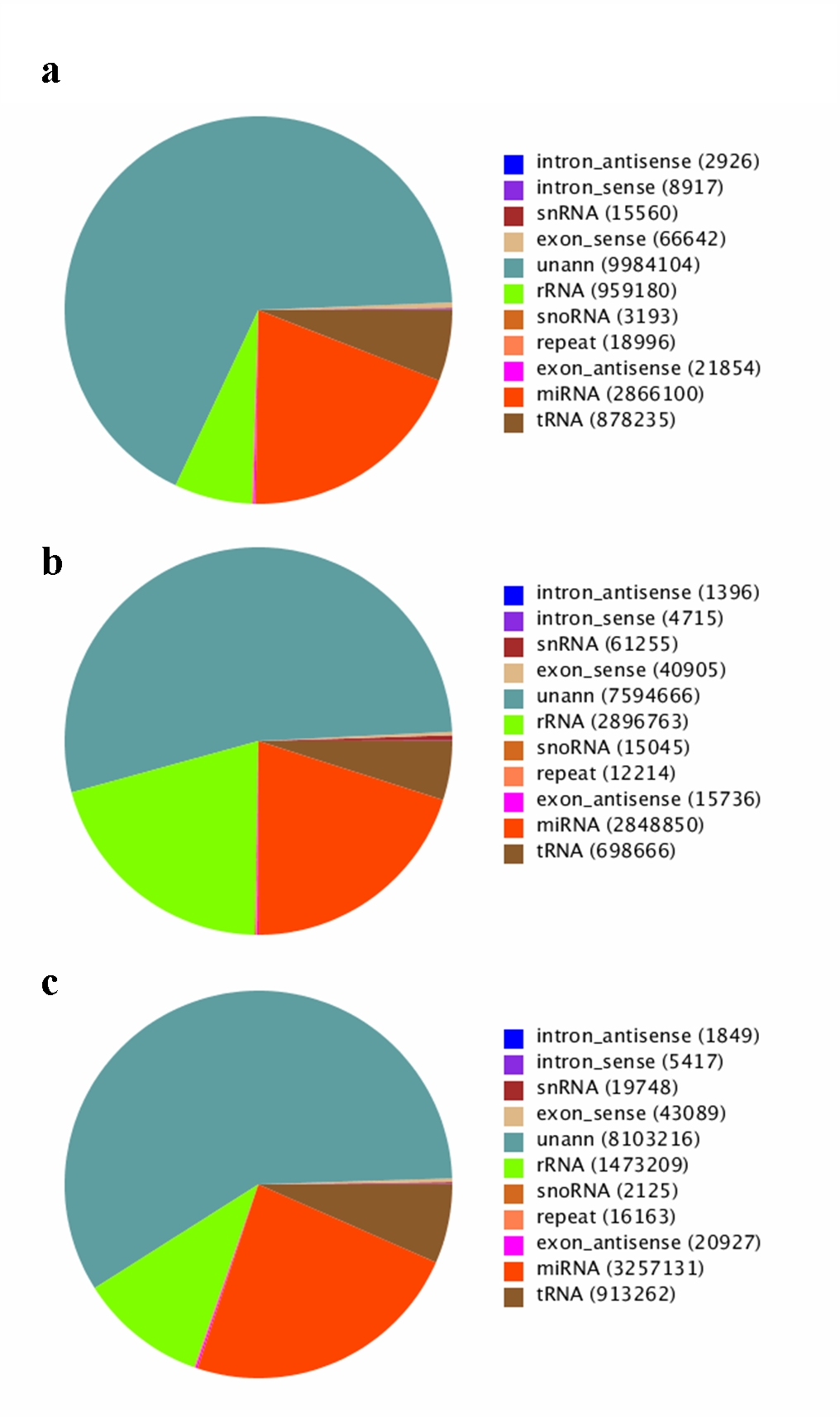

Supplement: Figure S1 — Read abundance of various classifications of small RNAs in the L. chinensis libraries. A: Read abundance of various classifications of small RNAs in the control sample, B: Read abundance of various classifications of small RNAs in the saline-alkali stress sample C: Read abundance of various classifications of small RNAs in the drought stress sample. (TIF) [file pone.0105417.s001.tif]
