# Supplementary material for: Discovery and Analysis of MicroRNAs in Leymus chinensis under Saline-Alkali and Drought Stress Using High-Throughput Sequencing
Source: PLoS One. 2014 Nov 4;9(11):e105417. doi: 10.1371/journal.pone.0105417 (PMC4219666; doi:10.1371/journal.pone.0105417)
Supplement: Table S1 — microRNA primers for qRT-PCR. (DOC) [file pone.0105417.s002.doc]

Table S1: microRNA primers for qRT-PCR

| microRNA primer | sequence 5'-3' |
| --- | --- |
| lch-miR160a | TGCCTGGCTCCCTGTATGCCA |
| lch-miR169f | TAGCCAAGGATGACTTGCCTA |
| lch-miR172d | AGAATCTTGATGATGCTGCAT |
| lch-miR319a | TTGGACTGAAGGGTGCTCCC |
| lch-miR394 | TTGGCATTCTGTCCACCTCC |
| lch-miR396f-3p | ATAGTTCAAGAGTCCTTGGAAA |
| lch-miR397a | TTGAGTGCAGCGTTGATGAAC |
| 5s rRNA | GGAAAAATAGCTCGGCGCCA |
