# Supplementary material for: Discovery and Analysis of MicroRNAs in Leymus chinensis under Saline-Alkali and Drought Stress Using High-Throughput Sequencing
Source: PLoS One. 2014 Nov 4;9(11):e105417. doi: 10.1371/journal.pone.0105417 (PMC4219666; doi:10.1371/journal.pone.0105417)
Supplement: Table S2 — microRNA and unigene primers for qRT-PCR. (DOC) [file pone.0105417.s003.doc]

Table S2: microRNA and unigene primers for qRT-PCR

| microRNA primer | sequence 5'-3' |
| --- | --- |
| lch-miR164c | GGAGAAGCAGGGTACGTGC |
| lch-miR172b | GGAATCTTGATGATGCTGCAT |
| lch-miR444a | GCAGTTGCTGCCTCAAGCTT |
| lch-miR1120 | TCTTATATTATGGGACGGAGG |
| lch-miR1318 | ATCAGGAGAGATGACACCGAC |
| GW_c54682F | CCTCAGAGGGGCCAGATTAG |
| GW_c54682R | GTCGGTTTATCAGTGTGGCC |
| GW_rep_c83825F | ATTTACCATCATCGGCTCGC |
| GW_rep_c83825R | ATGTTGGGCCTTTTGTTCGT |
| GW_rep_c1397F | TAGCTCCAACACCACGGAAT |
| GW_rep_c1397R | TGCCAAATTTCGTCATGCCA |
| GW_rep_c68861F | TCGATCCGAGATTCATGGCA |
| GW_rep_c68861R | CTAGCATTTCTCGACACGCC |
| GW_rep_c532F | ATGCCTAGAGACACCACCAC |
| GW_rep_c532R | GTTTGAAGATCCCCGTGACG |
| GW_rep_c3556F | CATGAAGATGAACCCGCTCG |
| GW_rep_c3556R | CCATTCCTGTAAGTGCTGCC |
| GW_rep_c66740F | TGAGGCAGCACTTACAGGAA |
| GW_rep_c66740R | AAAACCTCAGCAGCAATGGG |
| GW_rep_c57130F | GCGGCGATTAGTTGTTGCTA |
| GW_rep_c57130R | GCTGAGTTTCGAAAAGGCCA |
| GW_rep_c57893F | AGCCCAAACATAGCCTCCTT |
| GW_rep_c57893R | CTGCGGCTTCTATGGCAATT |
| GW_rep_c7398F | GAGCAGGCCTTCTCCAAGTA |
| GW_rep_c7398R | GTTGACGGTGATGTTGCGG |
| GW_rep_c1386F | TGCTTCCGTTCCTTCTGAGT |
| GW_rep_c1386R | AACTGATGGGTGGAGAGGTG |
| GW_rep_c30852F | CGCTGGCCTTCATCATCTTC |
| GW_rep_c30852R | AGAAGCACCACAATCTCCCA |
| GW_rep_c7381F | TCCGTTCCGTCTTCTTCTCC |
| GW_rep_c7381R | CAACTCCCAACAGCAAGGAC |
