# Supplementary material for: Discovery and Analysis of MicroRNAs in Leymus chinensis under Saline-Alkali and Drought Stress Using High-Throughput Sequencing
Source: PLoS One. 2014 Nov 4;9(11):e105417. doi: 10.1371/journal.pone.0105417 (PMC4219666; doi:10.1371/journal.pone.0105417)
Supplement: Table S3 — 5′ RACE PCR GSP primer. (DOC) [file pone.0105417.s004.doc]

Table S3 5’ RACE PCR GSP primer

| Primer name | sequence |
| --- | --- |
| GW_c13268 GSP1 primer | GTCTACACCGTCGAGGAGAT |
| GW_c13268 GSP2 primer | CCAAGGTCTTCTCCCGCAT |
|  |  |
| GW_rep_c1397 GSP1 primer | TTATTGTAGTGCGAGCCGATGAT |
| GW_rep_c1397 GSP2 primer | TCCCACGACTTAATTTTGGACC |
|  |  |
| GW_rep_c532 GSP1 primer | TTCATCCCTTCCGAACAGCT |
| GW_rep_c532 GSP2 primer | GGGAGGTGGAGGATCTGTTC |
|  |  |
| GW_rep_c3556 GSP1 primer | AGGTCCCATTGATGAGCACA |
| GW_rep_c3556 GSP2 primer | CTAGCACCTCTCAGTCTCCC |
|  |  |
| GW_rep_c57893 GSP1 primer | ACCGTGTTCAGAGAGCCAAT |
| GW_rep_c57893 GSP2 primer | ACCGTGTTCAGAGAGCCAAT |
|  |  |
| GW_rep_c1386 GSP1 primer | GTTCTCGGTTGACTGCTGTT |
| GW_rep_c1386 GSP2 primer | TGAGGGTGGCATCAGTAACA |
|  |  |
| GW_rep_c7381 GSP1 primer | TCCAATGGAAAGGGCAAACG |
| GW_rep_c7381 GSP2 primer | TGGGTTGAAGAATGTGCGAC |
|  |  |
