# Supplementary material for: Discovery and Analysis of MicroRNAs in Leymus chinensis under Saline-Alkali and Drought Stress Using High-Throughput Sequencing
Source: PLoS One. 2014 Nov 4;9(11):e105417. doi: 10.1371/journal.pone.0105417 (PMC4219666; doi:10.1371/journal.pone.0105417)
Supplement: Table S4 — Known miRNAs in the three small RNA libraries. (DOC) [file pone.0105417.s005.doc]

**Table S4:** Known miRNAs in three small RNA libraries.

| miRNA Family | miRNA ID | sequence | length | reference miRNA | control | saline-alkali | drought |
| --- | --- | --- | --- | --- | --- | --- | --- |
| miR156 | miR156a | TGACAGAAGAGAGTGAGCAC | 20 | osa-miR156a,e,i | 1846624 | 1982109 | 2161150 |
|  | miR156b | TGACAGAAGAGAGTGAGCAC | 20 | osa-miR156b | 1879080 | 2023323 | 2201075 |
|  | miR156d | TGACAGAAGAGAGTGAGCAC | 20 | osa-miR156d | 2039447 | 2147423 | 2347874 |
|  | miR156g | TGACAGAAGAGAGTGAGCAC | 20 | osa-miR156g | 1878825 | 2023026 | 2200704 |
|  | miR156h | TGACAGAAGAGAGTGAGCAC | 20 | osa-miR156h,j | 2038713 | 2146841 | 2347086 |
|  | miR156k | TGACAGAAGAGAGAGAGCAC | 20 | osa-miR156k | 1305 | 1611 | 1650 |
|  | miR156l | CGACAGAAGAGAGTGAGCA | 19 | osa-miR156l | 28 | 26 | 48 |
| miR159 | miR159a | TTTGGATTGAAGGGAGCT | 18 | hvu-miR159a,b | 12528 | 6258 | 10035 |
|  | miR159c | TTGGATTGAAGGGAGCTC | 18 | osa-miR159c,d,e | 11 | 6 | 24 |
|  | miR159f | CTTGGATTGAAGGGAGCTCT | 20 | osa-miR159f | 407 | 384 | 553 |
| miR160 | miR160a | TGCCTGGCTCCCTGTATGCCA | 21 | osa-miR160a,b,c | 35 | 18 | 271 |
|  | miR160d | TGCCTGGCTCCCTGTATGCCA | 21 | osa-miR160d | 42 | 22 | 285 |
|  | miR160e | ATGCCTGGCTCCCTGTATGCC | 21 | osa-miR160e | 0 | 1 | 3 |
|  | miR160f | TTGGCATTGAGGGAGTCATGC | 21 | osa-miR160f | 31 | 11 | 175 |
| miR162 | miR162a | TCGATAAACCTCTGCATCCAG | 21 | osa-miR162a | 0 | 0 | 3 |
| miR164 | miR164a | TGGAGAAGCAGGGCACGTGCA | 21 | osa-miR164a | 8600 | 6294 | 9026 |
|  | miR164b | TGGAGAAGCAGGGCACGTGC | 20 | osa-miR164b | 8605 | 6301 | 9027 |
|  | miR164c | TGGAGAAGCAGGGTACGTGCA | 21 | osa-miR164c | 4 | 1 | 1 |
|  | miR164d | TCGCTTGGTGCAGATCGGGAC | 21 | osa-miR164d | 1248 | 1018 | 1015 |
|  | miR164e | TGGAGAAGCAGGGCACGTG | 19 | osa-miR164e | 19 | 20 | 27 |
|  | miR164f | TGGAGAAGCAGGGCACGTGCA | 21 | osa-miR164f | 8607 | 6303 | 9027 |
| miR166 | miR166 | TCGGACCAGGCTTCATTCCCC | 21 | hvu-miR166 | 88293 | 66517 | 56848 |
|  | miR166a | TCGGACCAGGCTTCATTCCCC | 21 | osa-miR166a,d,f | 79528 | 59454 | 46661 |
|  | miR166b | TCGGACCAGGCTTCATTCCCC | 21 | osa-miR166b | 88193 | 66440 | 56774 |
|  | miR166c | TCGGACCAGGCTTCATTCCCC | 21 | osa-miR166c | 79404 | 59375 | 46603 |
|  | miR166e | TCGAACCAGGCTTCATTCCCC | 21 | osa-miR166e | 88214 | 66457 | 56784 |
|  | miR166g | TCGGACCAGGCTTCATTCC | 19 | osa-miR166g | 9 | 10 | 6 |
|  | miR166h | TCGGACCAGGCTTCATTCC | 19 | osa-miR166h | 680 | 523 | 946 |
|  | miR166k | TCGGACCAGGCTTCAATCCCT | 21 | osa-miR166k | 677 | 516 | 936 |
|  | miR166l | TCGGACCAGGCTTCAATCCC | 21 | osa-miR166l | 37652 | 23582 | 26294 |
|  | miR166m | TCGGACCAGGCTTCATTCCCT | 21 | osa-miR166m | 37673 | 23599 | 26313 |
|  | miR166n | TCGGACCAGGCTTCATTCCCC | 21 | osa-miR166n | 88279 | 66508 | 56829 |
| miR167 | miR167a | TGAAGCTGCCAGCATGATCTA | 21 | osa-miR167a,c | 11710 | 9325 | 11369 |
|  | miR167b | TGAAGCTGCCAGCATGATCTA | 21 | osa-miR167b | 11718 | 9329 | 11420 |
|  | miR167d | TGAAGCTGCCAGCATGATCTGA | 22 | osa-miR167d | 50567 | 69676 | 67525 |
|  | miR167e | TGAAGCTGCCAGCATGATCTG | 21 | osa-miR167e | 610 | 862 | 1026 |
|  | miR167f | TGAAGCTGCCAGCATGATCTGA | 22 | osa-miR167f | 49630 | 68612 | 66050 |
|  | miR167g | TGAAGCTGCCAGCATGATCTGA | 22 | osa-miR167g | 50258 | 69411 | 67000 |
|  | miR167h | TGAAGCTGCCAGCATGATCTGA | 22 | osa-miR167h | 49628 | 68618 | 66050 |
|  | miR167i | CTGAAGCTGCCAGCATGATCTG | 22 | osa-miR167i | 1519 | 1909 | 2477 |
|  | miR167j | TGAAGCTGCCAGCATGATCTGA | 22 | osa-miR167j | 50570 | 69676 | 67526 |
| miR168 | miR168-5p | TCGCTTGGTGCAGATCGGGAC | 21 | hvu-miR168-5p | 471865 | 409609 | 565693 |
|  | miR168a | TCGCTTGGTGCAGATCGGGAC | 21 | osa-miR168a | 471837 | 409594 | 565675 |
|  | miR168a-3p | TCGCTTGGTGCAGATCGGGAC | 21 | osa-miR168a-3p | 88 | 93 | 88 |
| miR169 | miR169a | CAGCCAAGGATGACTTGCCGA | 21 | osa-miR169a | 115 | 77 | 64 |
|  | miR169b | CAGCCAAGGATGACTTGCCGG | 21 | osa-miR169b | 287 | 164 | 197 |
|  | miR169c | CAAGGATGACTTGCCGGCTCC | 21 | osa-miR169c | 440 | 238 | 331 |
|  | miR169e | TAGCCAAGGATGACTTGCCG | 20 | osa-miR169e | 66 | 32 | 81 |
|  | miR169f | TAGCCAAGGATGACTTGCCTA | 21 | osa-miR169f | 17 | 7 | 41 |
|  | miR169g | GGCAGTCTCCTTGGCTAGC | 19 | osa-miR169g | 16 | 7 | 40 |
|  | miR169k | TAGCCAAGGATGACTTGCCTG | 21 | osa-miR169k,l | 59 | 35 | 83 |
|  | miR169m | TAGCCAAGGATGACTTGCCTG | 21 | osa-miR169m,i,j,h | 65 | 38 | 82 |
|  | miR169n | TAGCCAAGAATGACTTGCCT | 20 | osa-miR169n | 7 | 3 | 20 |
| miR171 | miR171a | TGATTGAGCCGCGCCAATATC | 21 | osa-miR171a | 793 | 597 | 539 |
|  | miR171b | TGATTGAGCCGTGCCAATATC | 21 | osa-miR171b,c | 131 | 57 | 69 |
|  | miR171d | TGATTGAGCCGTGCCAATATC | 21 | osa-miR171d | 819 | 601 | 560 |
|  | miR171e | TGATTGAGCCGTGCCAATATC | 21 | osa-miR171e | 795 | 596 | 541 |
|  | miR171f | TGATTGAGCCGTGCCAATATC | 21 | osa-miR171f | 793 | 597 | 539 |
|  | miR171h | TGGTATTGTTTCGGCTCATG | 20 | osa-miR171h | 820 | 603 | 565 |
|  | miR171i | TTGAGCCGCGTCAATATCTC | 20 | osa-miR171i | 15 | 51 | 50 |
| miR172 | miR172a | AGAATCTTGATGATGCTGCAT | 21 | osa-miR172a | 10755 | 6738 | 7897 |
|  | miR172b | GGAATCTTGATGATGCTGCAT | 21 | osa-miR172b | 53 | 41 | 33 |
|  | miR172c | GAATCTTGATGATGCTGCA | 19 | osa-miR172c | 1 | 0 | 0 |
|  | miR172d | AGAATCTTGATGATGCTGCAT | 21 | osa-miR172d | 10757 | 6738 | 7902 |
| miR319 | miR319a | ACTGGATGACGCGGGAGCTAA | 21 | osa-miR319a | 165 | 49 | 29 |
|  | miR319b | AGTGAATGAAGCGGGAGGTAA | 21 | osa-miR319b | 155 | 61 | 28 |
| miR390 | miR390 | AAGCTCAGGAGGGATAGCGCC | 21 | osa-miR390 | 344 | 151 | 118 |
| miR393 | miR393 | TCCAAAGGGATCGCATTGATC | 21 | osa-miR393 | 136 | 36 | 126 |
|  | miR393b | TCCAAAGGGATCGCATTGATC | 21 | osa-miR393b | 139 | 38 | 118 |
|  | miR393b-3p | TCCAAAGGGATCGCATTGATC | 21 | osa-miR393b-3p | 7 | 1 | 5 |
| miR394 | miR394 | TTGGCATTCTGTCCACCTCC | 20 | osa-miR394 | 30 | 13 | 122 |
| miR395 | miR395a | TGAAGTGCTTGGGGGAACTC | 20 | osa-miR395a | 0 | 1 | 1 |
|  | miR395b | TGAAGTGTTTGGGGGAACTC | 20 | osa-miR395e,i,k,j,b | 168 | 200 | 203 |
|  | miR395d | TGAAGTGTTTGGGGGAACTC | 20 | osa-miR395d | 169 | 200 | 203 |
|  | miR395g | TGAAGTGTTTGGGGGAACTC | 20 | osa-miR395g | 172 | 201 | 213 |
|  | miR395h | TGAAGTGTTTGGGGGAACTC | 20 | osa-miR395h | 166 | 200 | 203 |
|  | miR395l | TGAAGTGTTTGGGGGAACTC | 20 | osa-miR395l,n | 166 | 190 | 202 |
|  | miR395m | TGAAGTGTTTGGGGGAACTC | 20 | osa-miR395p,m | 162 | 189 | 192 |
|  | miR395q | TGAAGTGTTTGGGGGAACTC | 20 | osa-miR395q,r | 163 | 189 | 192 |
|  | miR395t | TGAAGTGTTTGGGGAAACTC | 20 | osa-miR395t | 1 | 0 | 0 |
| miR396 | miR396a | TTCCACAGCTTTCTTGAACTG | 21 | osa-miR396a,b | 290 | 198 | 234 |
|  | miR396c | TTCCACAGCTTTCTTGAACTT | 21 | osa-miR396c | 1742 | 756 | 895 |
|  | miR396d | TCCACAGGCTTTCTTGAACTG | 21 | osa-miR396d,e | 11152 | 7165 | 9260 |
|  | miR396e-3p | TCCACAGGCTTTCTTGAACTG | 21 | osa-miR396e-3p | 38 | 16 | 14 |
|  | miR396f | TCCACAGGCTTTCTTGAACTG | 21 | osa-miR396f | 11186 | 7171 | 9278 |
|  | miR396f-3p | TCCACAGGCTTTCTTGAACTG | 21 | osa-miR396f-3p | 26 | 5 | 7 |
|  | miR396g | TCCACAGGCTTTCTTGAAC | 19 | osa-miR396g,h,i, | 512 | 227 | 450 |
| miR397 | miR397 | TTGAGTGCAGCGTTGATGAAC | 21 | hvu-miR397 | 3413 | 3083 | 3654 |
|  | miR397a | TTGAGTGCAGCGTTGATGAAC | 21 | osa-miR397a | 3436 | 3108 | 3712 |
|  | miR397b | TTGAGTGCAGCGTTGATGAAC | 21 | osa-miR397b | 3426 | 3102 | 3690 |
| miR398 | miR398b | TGTGTTCTCAGGTCGCCC | 18 | osa-miR398b | 1 | 2 | 3 |
| miR399 | miR399 | TGCCAAAGGAGATTTGCCCCGAA | 23 | hvu-miR399 | 0 | 1 | 2 |
|  | miR399a | TGCCAAAGGAGAATTGCCCTG | 21 | osa-miR399a,b | 1 | 0 | 2 |
|  | miR399c | TGCCAAAGGAGAATTGCCCTG | 21 | osa-miR399c | 1 | 0 | 2 |
|  | miR399d | TGCCAAAGGAGAGTTGCCCTG | 21 | osa-miR399d | 6 | 1 | 3 |
|  | miR399f | TCTGCCAAAGGAGATTTGCCC | 21 | osa-miR399f,g | 3 | 0 | 0 |
|  | miR399j | TGCCAAAGGAGAGTTGCCC | 19 | osa-miR399j | 0 | 0 | 1 |
| miR408 | miR408 | TGCACTGCCTCTTCCCTGGC | 20 | osa-miR408 | 9 | 7 | 23 |
| miR437 | miR437 | ACTTGGTCAAAGTTAGAGAAGTTT | 24 | osa-miR437 | 0 | 0 | 1 |
| miR444a | miR444a | TGCAGTTGCTGCCTCAAGCTT | 21 | hvu-miR444a | 397 | 246 | 549 |
|  | miR444a.2 | TGCAGTTGCTGCCTCAAGCTT | 21 | osa-miR444a.2 | 401 | 247 | 553 |
|  | miR444b | TGCAGTTGCTGTCTCAAGCTT | 21 | hvu-miR444b | 118 | 83 | 107 |
|  | miR444b.1 | TGCAGTTGTTGTCTCAAGCTT | 21 | osa-miR444b.1,c.1 | 162 | 145 | 127 |
|  | miR444b.2 | TGCAGTTGTTGTCTCAAGCTT | 21 | osa-miR444b.2 | 228 | 210 | 194 |
|  | miR444c.2 | TGCAGTTGTTGTCTCAAGCTT | 21 | osa-miR444c.2 | 208 | 186 | 177 |
|  | miR444d.2 | TGCAGTTGCTGCCTCAAGCTT | 21 | osa-miR444d.2 | 402 | 247 | 552 |
|  | miR444d.3 | TGCAGTTGCTGCCTCAAGCTT | 21 | osa-miR444d.3 | 0 | 0 | 1 |
|  | miR444e | TGCAGTTGCTGCCTCAAGCTT | 21 | osa-miR444e | 405 | 249 | 555 |
|  | miR444f | TTTCTTGCAAGTTGTGCAGTT | 21 | osa-miR444f | 0 | 1 | 0 |
| miR528 | miR528 | TGGAAGGGGCATGCAGAGGA | 20 | osa-miR528 | 25851 | 14677 | 49071 |
| miR530 | miR530-5p | CTGCATTTGCACCTGCACCTA | 21 | osa-miR530-5p | 4 | 0 | 0 |
| miR535 | miR535 | TGACAACGAGAGAGAGCACGC | 21 | osa-miR535 | 2 | 1 | 0 |
| miR818 | miR818a | TTATATTATGGGACGGAAGG | 20 | osa-miR818a | 0 | 1 | 3 |
|  | miR818b | TTATATTATGGGACGGAGG | 19 | osa-miR818b,e | 15 | 6 | 11 |
|  | miR818c | TTATATTATGGGACGGATG | 19 | osa-miR818c | 2 | 0 | 1 |
|  | miR818d | CTTATATTATGGGACGGAGGGAGT | 24 | osa-miR818d | 306 | 194 | 257 |
| miR827 | miR827a | TTAGATGACCATCAGCAAACA | 21 | osa-miR827a | 1017 | 452 | 858 |
|  | miR827b | TTAGATGACCATCAGCAAACA | 21 | osa-miR827b | 1035 | 456 | 876 |
| miR1120 | miR1120 | TCTTATATTATGGGACGGAGG | 21 | hvu-miR1120 | 280 | 207 | 317 |
| miR1126 | miR1126 | ACTATGGACTACATACGGAACAAA | 24 | hvu-miR1126 | 32 | 23 | 18 |
| miR1318 | miR1318 | ATCAGGAGAGATGACACCGAC | 21 | osa-miR1318,1432 | 559 | 427 | 796 |
| miR1436 | miR1436 | ACATTATGGGACGGAGGGAGT | 21 | osa-miR1436 | 16 | 7 | 12 |
| miR1439 | miR1439 | TTTGGAACGGAGTGAGTAT | 19 | osa-miR1439 | 0 | 0 | 1 |
| miR5048 | miR5048 | TTTGCAGGTTTTAGGTCTAAGT | 22 | hvu-miR5048 | 20935 | 11194 | 20608 |
| miR5050 | miR5050 | TTTTGCCGGTTGAACGACCTCA | 22 | hvu-miR5050 | 2 | 0 | 1 |
| miR5072 | miR5072 | TCCCCAGCGGAGTCGCCA | 18 | osa-miR5072 | 5 | 5 | 11 |
| miR5077 | miR5077 | TTCGCGTCGGGTTCACCA | 18 | osa-miR5077 | 0 | 0 | 1 |
| miR5083 | miR5083 | AGACTACAATTATCTGATCA | 20 | osa-miR5083 | 19 | 25 | 11 |
