# Supplementary material for: Discovery and Analysis of MicroRNAs in Leymus chinensis under Saline-Alkali and Drought Stress Using High-Throughput Sequencing
Source: PLoS One. 2014 Nov 4;9(11):e105417. doi: 10.1371/journal.pone.0105417 (PMC4219666; doi:10.1371/journal.pone.0105417)
Supplement: Table S5 — Target genes of known miRNAs. (DOCX) [file pone.0105417.s006.docx]

| Table S5: Target genes of known miRNAs | | |  |  |
| --- | --- | --- | --- | --- |
| microRNAs | Target unigene | score | Inhibition |  |
| lch-miR156a,b,d,g,h,k | GW_rep_c24563 | 1 | Cleavage | RNA recognition motif containing protein |
| lch-miR156a,b,d,g,h,k | GW_c18971 | 2 | Cleavage | ATPase involved in DNA repair |
| lch-miR156a,b,d,g,h,k | GW_rep_c14188 | 3 | translation | Cytochrome P450 |
| lch-miR156a,b,d,g,h,k | GW_rep_c4101 | 3 | Cleavage | Actin and related proteins |
| lch-miR156a,b,d,g,h,k | GW_rep_c4201 | 3.5 | Cleavage | Molecular chaperone |
| lch-miR156l | GW_c18971 | 2 | Cleavage | ATPase involved in DNA repair |
| lch-miR156l | GW_rep_c4497 | 3.5 | Cleavage | Serine/threonine protein kinase |
| lch-miR159f | GW_rep_c108567 | 3 | Cleavage | Ribose 5-phosphate isomerase |
| lch-miR159f | GW_rep_c80136 | 3 | Cleavage | FAD/FMN-containing dehydrogenases |
| lch-miR159f | GW_rep_c13442 | 3 | translation | Enolase |
| lch-miR159f | GW_c7103 | 3.5 | Cleavage | Cation transport ATPase |
| lch-miR160e | GW_rep_c26347 | 3.5 | Cleavage | ATP-dependent 26S proteasome regulatory subunit |
| lch-miR160f | GW_rep_c12122 | 3 | Cleavage | ATPases with chaperone activity |
| lch-miR160f | GW_rep_c3189 | 3 | Cleavage | Acetyl-CoA carboxylase |
| lch-miR162a | GW_rep_c66261 | 3 | Cleavage | Putative NADP-dependent oxidoreductases |
| lch-miR162a | GW_rep_c48132 | 3 | Cleavage | Putative NADP-dependent oxidoreductases |
| lch-miR162a | GW_rep_c57184 | 3 | Cleavage | Putative NADP-dependent oxidoreductases |
| lch-miR162a | GW_rep_c61430 | 3 | Cleavage | Putative NADP-dependent oxidoreductases |
| lch-miR162a | GW_rep_c75375 | 3 | Cleavage | Putative NADP-dependent oxidoreductases |
| lch-miR162a | GW_c18807 | 4 | translation | Ubiquitin-protein ligase |
| lch-miR164c | GW_c54682 | 2.5 | Cleavage | Phosphoribosylformylglycinamidine (FGAM) synthase |
| lch-miR164f | GW_rep_c15227 | 3.5 | Cleavage | Geranylgeranyl pyrophosphate synthase |
| lch-miR164a | GW_c13268 | 3.5 | Cleavage | Molecular chaperone |
| lch-miR164f | GW_rep_c55320 | 3.5 | translation | Phospholipase C |
| lch-miR169c | GW_c25032 | 1.5 | Cleavage | Acetyltransferases |
| lch-miR169c | GW_c25836 | 2.5 | Cleavage | Secreted/periplasmic Zn-dependent peptidases, insulinase-like |
| lch-miR169c | GW_rep_c4812 | 3 | translation | Tubulin |
| lch-miR169c | GW_rep_c35446 | 3 | translation | Dinucleotide-utilizing enzymes involved in molybdopterin and thiamine  biosynthesis family 2 |
| lch-miR169c | GW_rep_c82274 | 3 | translation | Dinucleotide-utilizing enzymes involved in molybdopterin and thiamine  biosynthesis family 2 |
| lch-miR169n | GW_rep_c12498 | 3 | Cleavage | Malate/lactate dehydrogenases |
| lch-miR169o | GW_rep_c1791 | 3.5 | translation | ATPases of the AAA+ class |
| lch-miR169n | GW_rep_c22775 | 3.5 | Cleavage | Predicted aminopeptidases |
| lch-miR169o | GW_c4442 | 3.5 | Cleavage | Predicted aminopeptidases |
| lch-miR393 | GW_rep_c13932 | 3 | Cleavage | Coproporphyrinogen III oxidase |
| lch-miR393b | GW_rep_c77809 | 3.5 | Cleavage | Predicted nucleoside-diphosphate-sugar epimerases |
| lch-miR393b-3p | GW_rep_c58284 | 3.5 | Cleavage | Predicted nucleoside-diphosphate-sugar epimerases |
| lch-miR393 | GW_rep_c9149 | 4 | Cleavage | U2 snRNP spliceosome subunit |
| lch-miR393b | GW_c33530 | 3.5 | translation | Predicted metal-dependent membrane protease |
| lch-miR396d | GW_rep_c9283 | 3.5 | Cleavage | Glutamyl- and glutaminyl-tRNA synthetases |
| lch-miR396e-3p | GW_c82395 | 3.5 | Cleavage | Glutamyl- and glutaminyl-tRNA synthetases |
| lch-miR396e-3p | GW_rep_c49343 | 3.5 | Cleavage | UDP-glucose 4-epimerase |
| lch-miR319a | GW_rep_c24088 | 2 | translation | 26S proteasome regulatory complex component |
| lch-miR319a | GW_rep_c74568 | 3 | Cleavage | 26S proteasome regulatory complex component |
| lch-miR319a | GW_rep_c58089 | 3.5 | Cleavage | Ribulose bisphosphate carboxylase small subunit |
| lch-miR172a | GW_rep_c83825 | 2 | Cleavage | RNA-binding proteins |
| lch-miR172d | GW_rep_c1397 | 2 | Cleavage | RNA-binding proteins |
| lch-miR172a | GW_rep_c74528 | 2.5 | Cleavage | Succinyl-CoA synthetase, beta subunit |
| lch-miR172d | GW_rep_c1412 | 2.5 | Cleavage | Succinyl-CoA synthetase, beta subunit |
| lch-miR172a | GW_rep_c6753 | 2.5 | Cleavage | Succinyl-CoA synthetase, beta subunit |
| lch-miR172d | GW_c44242 | 3 | Cleavage | ABC-type multidrug transport system |
| lch-miR172a | GW_rep_c34913 | 3 | Cleavage | HSP60 family |
| lch-miR172d | GW_c24605 | 3.5 | Cleavage | DNA-directed RNA polymerase |
| lch-miR172a | GW_rep_c68861 | 3.5 | translation | DnaJ-class molecular chaperone with C-terminal Zn finger domain |
| lch-miR319b | GW_rep_c1248 | 3 | Cleavage | NAD-dependent aldehyde dehydrogenases |
| lch-miR319b | GW_c48003 | 3 | Cleavage | Glycosyltransferases |
| lch-miR319b | GW_c108184 | 3 | Cleavage | Glycosyltransferases |
| lch-miR319b | GW_rep_c59844 | 3.5 | Cleavage | NAD-dependent aldehyde dehydrogenases |
| lch-miR319b | GW_rep_c107675 | 3.5 | Cleavage | NAD-dependent aldehyde dehydrogenases |
| lch-miR172b | GW_rep_c83825 | 2.5 | Cleavage | RNA-binding proteins |
| lch-miR172b | GW_rep_c1397 | 2.5 | Cleavage | RNA-binding proteins |
| lch-miR172b | GW_rep_c68861 | 2.5 | translation | DnaJ-class molecular chaperone with C-terminal Zn finger domain |
| lch-miR172b | GW_rep_c532 | 3 | Cleavage | RNA-binding proteins |
| lch-miR172b | GW_rep_c74528 | 3 | Cleavage | Succinyl-CoA synthetase |
| lch-miR172b | GW_rep_c1412 | 3 | Cleavage | Succinyl-CoA synthetase |
| lch-miR172b | GW_rep_c6753 | 3 | Cleavage | Succinyl-CoA synthetase |
| lch-miR172b | GW_rep_c34913 | 3.5 | Cleavage | Chaperonin GroEL |
| lch-miR171h | GW_c44994 | 3 | Cleavage | Serine/threonine protein kinase |
| lch-miR171h | GW_rep_c101396 | 3 | Cleavage | Acetyl-CoA acetyltransferase |
| lch-miR171h | GW_rep_c21662 | 3 | Cleavage | Acetyl-CoA acetyltransferase |
| lch-miR171h | GW_rep_c86308 | 3 | Cleavage | Acetyl-CoA acetyltransferase |
| lch-miR437 | GW_rep_c53155 | 3 | Cleavage | 3-phosphoglycerate kinase |
| lch-miR437 | GW_rep_c1506 | 3.5 | translation | Ubiquitin-protein ligase |
| lch-miR437 | GW_rep_c201 | 3.5 | translation | Ubiquitin-protein ligase |
| lch-miR818d | GW_c9898 | 0 | Cleavage | RNA-binding proteins |
| lch-miR818d | GW_rep_c18862 | 0 | Cleavage | Histidinol dehydrogenase |
| lch-miR818d | GW_rep_c106055 | 0 | Cleavage | Glutathione S-transferase |
| lch-miR818d | GW_rep_c7398 | 1 | Cleavage | Lignostilbene-alpha,beta-dioxygenase and related enzymes |
| lch-miR535 | GW_rep_c65506 | 3 | translation | Glutathione S-transferase |
| lch-miR535 | GW_c49847 | 3 | Cleavage | Myb superfamily proteins, including transcription factors and mRNA  splicing factors |
| lch-miR408 | GW_rep_c9470 | 2.5 | Cleavage | Phytoene/squalene synthetase |
| lch-miR408 | GW_c6008 | 2.5 | Cleavage | Phytoene/squalene synthetase |
| lch-miR408 | GW_rep_c36948 | 3 | Cleavage | NADH dehydrogenase |
| lch-miR444a | GW_c15584 | 1.5 | Cleavage | RING-finger-containing E3 ubiquitin ligase |
| lch-miR444a | GW_rep_c86536 | 3.5 | Cleavage | Malate/lactate dehydrogenases |
| lch-miR444a | GW_rep_c3556 | 3.5 | Cleavage | Malate/lactate dehydrogenases |
| lch-miR444a | GW_rep_c66740 | 3.5 | Cleavage | Malate/lactate dehydrogenases |
| lch-miR444a | GW_rep_c55950 | 3.5 | Cleavage | Malate/lactate dehydrogenases |
| lch-miR444a | GW_rep_c57130 | 3.5 | Cleavage | Malate/lactate dehydrogenases |
| lch-miR444a | GW_rep_c57893 | 3.5 | Cleavage | Isocitrate dehydrogenases |
| lch-miR444a | GW_rep_c62088 | 3.5 | Cleavage | Malate/lactate dehydrogenases |
| lch-miR444a | GW_rep_c1092 | 3.5 | Cleavage | Malate/lactate dehydrogenases |
| lch-miR444b | GW_c15584 | 2 | Cleavage | RING-finger-containing E3 ubiquitin ligase |
| lch-miR444b | GW_c10029 | 3 | Cleavage | Serine/threonine protein kinase |
| lch-miR444b.1 | GW_c15584 | 2.5 | Cleavage | RING-finger-containing E3 ubiquitin ligase |
| lch-miR444b.1 | GW_rep_c2155 | 3.5 | Cleavage | Predicted RNA-binding protein |
| lch-miR444b.1 | GW_c10029 | 3.5 | Cleavage | Serine/threonine protein kinase |
| lch-miR444b.1 | GW_c6928 | 3.5 | translation | Chromosome segregation ATPases |
| lch-miR528 | GW_rep_c54716 | 2 | translation | Enolase |
| lch-miR528 | GW_rep_c20652 | 2.5 | Cleavage | FAD/FMN-containing dehydrogenases |
| lch-miR528 | GW_rep_c82202 | 3 | translation | Cytosine/adenosine deaminases |
| lch-miR818a | GW_c9898 | 1 | Cleavage | RNA-binding proteins |
| lch-miR818a | GW_rep_c18862 | 1 | Cleavage | Histidinol dehydrogenase |
| lch-miR818a | GW_rep_c106055 | 2 | Cleavage | Glutathione S-transferase |
| lch-miR818a | GW_rep_c7398 | 2.5 | Cleavage | Lignostilbene-alpha,beta-dioxygenase and related enzymes |
| lch-miR444f | GW_rep_c50284 | 3 | Cleavage | GTPase SAR1 and related small G proteins |
| lch-miR444f | GW_rep_c7021 | 3.5 | Cleavage | Glycosyltransferases, probably involved in cell wall biogenesis |
| lch-miR1436 | GW_rep_c316 | 1.5 | Cleavage | Esterase/lipase |
| lch-miR1436 | GW_c9898 | 2.5 | Cleavage | RNA-binding proteins |
| lch-miR1436 | GW_rep_c106055 | 3 | Cleavage | Glutathione S-transferase |
| lch-miR5083 | GW_c11574 | 3.5 | Cleavage | Thioredoxin-like proteins and domains |
| lch-miR5083 | GW_rep_c87771 | 3.5 | Cleavage | Translation elongation factor EF-1beta |
| lch-miR1318 | GW_rep_c66483 | 3 | translation | Predicted membrane protein |
| lch-miR1318 | GW_c23219 | 3 | Cleavage | HrpA-like helicases |
| lch-miR1318 | GW_rep_c30852 | 3 | Cleavage | Thiol-disulfide isomerase and thioredoxins |
| lch-miR1318 | GW_rep_c7381 | 3.5 | translation | Predicted membrane protein |
| lch-miR1120 | GW_rep_c18862 | 0 | Cleavage | Histidinol dehydrogenase |
| lch-miR1120 | GW_c9898 | 0 | Cleavage | RNA-binding proteins |
| lch-miR1120 | GW_rep_c106055 | 1 | translation | Glutathione S-transferase |
| lch-miR1120 | GW_rep_c7398 | 1 | Cleavage | Lignostilbene-alpha,beta-dioxygenase and related enzymes |
| lch-miR1120 | GW_rep_c1386 | 2 | Cleavage | Signal recognition particle GTPase |
| lch-miR5048 | GW_c34555 | 1 | Cleavage | Serine/threonine protein kinase |
| lch-miR5048 | GW_c41075 | 2 | Cleavage | Serine/threonine protein kinase |
| lch-miR5050 | GW_rep_c105250 | 3 | translation | Predicted hydrolases or acyltransferases |
