# Supplementary material for: Discovery and Analysis of MicroRNAs in Leymus chinensis under Saline-Alkali and Drought Stress Using High-Throughput Sequencing
Source: PLoS One. 2014 Nov 4;9(11):e105417. doi: 10.1371/journal.pone.0105417 (PMC4219666; doi:10.1371/journal.pone.0105417)
Supplement: Table S6 — Target genes of novel miRNAs. (DOCX) [file pone.0105417.s007.docx]

| Table S6: Target genes of novel miRNAs | | | | |
| --- | --- | --- | --- | --- |
| **microRNAs** | **Target gene** | **score** | **Inhibition** | **Function description** |
| lch-MIR-03 | GW_rep_c902 | 3 | cleavage | Glycosyltransferases, probably involved in cell wall biogenesis |
| lch-MIR-03 | GW_rep_c84294 | 3 | cleavage | Glycosyltransferases, probably involved in cell wall biogenesis |
| lch-MIR-03 | GW_rep_c54049 | 3 | cleavage | Glyceraldehyde-3-phosphate dehydrogenase/erythrose-4-phosphate dehydrogenase |
| lch-MIR-03 | GW_rep_c62959 | 3 | cleavage | Glyceraldehyde-3-phosphate dehydrogenase/erythrose-4-phosphate dehydrogenase |
| lch-MIR-03 | GW_rep_c22910 | 3 | cleavage | Glyceraldehyde-3-phosphate dehydrogenase/erythrose-4-phosphate dehydrogenase |
| lch-MIR-08 | GW_rep_c51817 | 2.5 | cleavage | Aspartate/tyrosine/aromatic aminotransferase |
| lch-MIR-08 | GW_rep_c44647 | 2.5 | cleavage | Aspartate/tyrosine/aromatic aminotransferase |
| lch-MIR-08 | GW_rep_c14300 | 2.5 | cleavage | Aspartate/tyrosine/aromatic aminotransferase |
| lch-MIR-08 | GW_rep_c60338 | 2.5 | cleavage | Aspartate/tyrosine/aromatic aminotransferase |
| lch-MIR-08 | GW_rep_c95753 | 3 | cleavage | Ubiquitin |
| lch-MIR-08 | GW_c35967 | 3 | cleavage | Aspartate/tyrosine/aromatic aminotransferase |
| lch-MIR-08 | GW_rep_c49083 | 3 | cleavage | Aspartate/tyrosine/aromatic aminotransferase |
| lch-MIR-02 | GW_c33890 | 3 | Translation | F0F1-type ATP synthase |
| lch-MIR-13 | GW_c31294 | 3 | cleavage | Sulfite reductase |
| lch-MIR-13 | GW_rep_c30583 | 3.5 | cleavage | Glutathione S-transferase |
| lch-MIR-15 | GW_c9190 | 3 | cleavage | dihydrolipoamide dehydrogenase |
| lch-MIR-15 | GW_c19713 | 3.5 | Translation | Predicted rRNA methylase |
| lch-MIR-11 | GW_rep_c13299 | 1 | Translation | SAM-dependent methyltransferases |
| lch-MIR-11 | GW_rep_c19334 | 1 | cleavage | Ribulose bisphosphate carboxylase small subunit |
| lch-MIR-11 | GW_c7940 | 1.5 | Translation | Ribulose bisphosphate carboxylase small subunit |
| lch-MIR-11 | GW_rep_c55447 | 1.5 | Translation | Ribulose bisphosphate carboxylase small subunit |
| lch-MIR-11 | GW_rep_c71390 | 1.5 | Translation | Ribulose bisphosphate carboxylase small subunit |
| lch-MIR-11 | GW_rep_c80308 | 2 | Translation | Xanthine dehydrogenase, molybdopterin-binding subunit B |
| lch-MIR-11 | GW_rep_c77706 | 2 | Translation | Ribulose bisphosphate carboxylase small subunit |
| lch-MIR-11 | GW_rep_c104711 | 2 | Translation | Ribulose bisphosphate carboxylase small subunit |
| lch-MIR-11 | GW_c68811 | 2 | Translation | Ribulose bisphosphate carboxylase small subunit |
| lch-MIR-11 | GW_rep_c30205 | 2 | Translation | Ribulose bisphosphate carboxylase small subunit |
| lch-MIR-06 | GW_rep_c2588 | 1 | cleavage | NAD-dependent aldehyde dehydrogenases |
| lch-MIR-06 | GW_c33890 | 2 | cleavage | F0F1-type ATP synthase |
| lch-MIR-06 | GW_c18586 | 2.5 | cleavage | Fe-S oxidoreductases |
| lch-MIR-06 | GW_rep_c57470 | 3.5 | cleavage | ATP-dependent 26S proteasome regulatory subunit |
| lch-MIR-06 | GW_c11930 | 3.5 | cleavage | tRNA and rRNA cytosine-C5-methylases |
| lch-MIR-06 | GW_rep_c25103 | 3.5 | cleavage | CCAAT-binding factor |
| lch-MIR-06 | GW_c53067 | 3 | cleavage | Serine/threonine protein kinase |
| lch-MIR-06 | GW_c41177 | 3 | Translation | UDP-glucose 4-epimerase |
| lch-MIR-04 | GW_rep_c1275 | 3.5 | Translation | Micrococcal nuclease |
| lch-MIR-04 | GW_c19341 | 3.5 | cleavage | Succinate dehydrogenase/fumarate reductase |
| lch-MIR-04 | GW_rep_c35905 | 3.5 | cleavage | Ribosomal protein L35AE/L33A |
| lch-MIR-04 | GW_rep_c81318 | 3.5 | cleavage | Glyceraldehyde-3-phosphate dehydrogenase/erythrose-4-phosphate dehydrogenase |
| lch-MIR-01 | GW_rep_c18862 | 1 | cleavage | Histidinol dehydrogenase |
| lch-MIR-01 | GW_c9898 | 1 | cleavage | RNA-binding proteins |
| lch-MIR-01 | GW_rep_c106055 | 2 | Translation | Glutathione S-transferase |
| lch-MIR-01 | GW_rep_c7398 | 2 | cleavage | Lignostilbene-alpha,beta-dioxygenase and related enzymes |
| lch-MIR-01 | GW_rep_c30843 | 2 | Translation | Glycosyl transferases, related to UDP-glucuronosyltransferase |
| lch-MIR-05 | GW_rep_c11545 | 3.5 | Translation | Uncharacterized conserved protein |
| lch-MIR-05 | GW_c4799 | 3.5 | cleavage | Predicted epimerase, PhzC/PhzF homolog |
| lch-MIR-05 | GW_c6149 | 3.5 | cleavage | FOG: TPR repeat |
| lch-MIR-12 | GW_rep_c33123 | 2 | cleavage | ATPase components of ABC transporters with duplicated ATPase domains |
| lch-MIR-12 | GW_rep_c18765 | 3.5 | cleavage | NADPH-dependent oxidoreductases |
| lch-MIR-12 | GW_rep_c7994 | 3.5 | cleavage | Serine/threonine protein kinase |
| lch-MIR-16 | GW_rep_c8044 | 1 | cleavage | Ribosomal protein L1 |
| lch-MIR-16 | GW_rep_c85098 | 1 | cleavage | Demethylmenaquinone methyltransferase |
| lch-MIR-16 | GW_rep_c45391 | 2.5 | cleavage | Predicted membrane protein |
| lch-MIR-16 | GW_rep_c12245 | 2.5 | cleavage | Zn-dependent hydrolases, including glyoxylases |
| lch-MIR-16 | GW_rep_c46688 | 2.5 | cleavage | Glycerol uptake facilitator and related permeases |
| lch-MIR-16 | GW_rep_c42201 | 2.5 | cleavage | Subtilisin-like serine proteases |
| lch-MIR-16 | GW_rep_c6596 | 3 | cleavage | Acetolactate synthase |
