# Supplementary material for: Discovery and Analysis of MicroRNAs in Leymus chinensis under Saline-Alkali and Drought Stress Using High-Throughput Sequencing
Source: PLoS One. 2014 Nov 4;9(11):e105417. doi: 10.1371/journal.pone.0105417 (PMC4219666; doi:10.1371/journal.pone.0105417)
Supplement: Table S7 — Expression profile of target unigenes. (DOC) [file pone.0105417.s008.doc]

Figure S7: Target unigenes expression profile

| GeneID | Gene length | Control Expression | [saline-alkali](http://dict.youdao.com/search?q=saline-alkali&keyfrom=E2Ctranslation)Expression | log2 Ratio(sample/control) | Up or Down Regulation | P-value |
| --- | --- | --- | --- | --- | --- | --- |
| GW_rep_c4201 | 2462 | 14 | 13 | -0.106915203916512 | Down | 0.221606 |
| GW_rep_c4497 | 661 | 5 | 2 | -1.32192809488736 | Down | 0.104811 |
| GW_rep_c80136 | 501 | 0 | 2 | 10.9657842846621 | Up | 0.423152 |
| GW_rep_c13442 | 1060 | 3 | 5 | 0.736965594166206 | Up | 0.944606 |
| GW_c7103 | 2723 | 53 | 87 | 0.715023041285529 | Up | 0.552714 |
| GW_rep_c3189 | 1671 | 9 | 10 | 0.15200309344505 | Up | 0.513768 |
| GW_c54682 | 478 | 0 | 12 | 13.5507467853832 | Up | 0.00238812 |
| GW_rep_c15227 | 824 | 2 | 3 | 0.584962500721156 | Up | 0.92839 |
| GW_c13268 | 1682 | 2 | 12 | 2.58496250072116 | Up | 0.0505014 |
| GW_rep_c55320 | 331 | 0 | 5 | 12.2877123795495 | Up | 0.0895292 |
| GW_c25032 | 702 | 1 | 3 | 1.58496250072116 | Up | 0.660144 |
| GW_rep_c4812 | 831 | 4 | 10 | 1.32192809488736 | Up | 0.415668 |
| GW_rep_c35446 | 670 | 1 | 4 | 2 | Up | 0.454306 |
| GW_rep_c82274 | 386 | 1 | 2 | 1 | Up | 0.936496 |
| GW_rep_c12498 | 1238 | 3 | 5 | 0.736965594166206 | Up | 0.944606 |
| GW_rep_c1791 | 1884 | 9 | 24 | 1.41503749927884 | Up | 0.1333698 |
| GW_rep_c22775 | 718 | 2 | 5 | 1.32192809488736 | Up | 0.613742 |
| GW_c4442 | 1730 | 6 | 12 | 1 | Up | 0.590474 |
| GW_rep_c24088 | 1231 | 3 | 6 | 1 | Up | 0.744018 |
| GW_rep_c74568 | 818 | 1 | 2 | 1 | Up | 0.936496 |
| GW_rep_c58089 | 421 | 0 | 2 | 10.9657842846621 | Up | 0.423152 |
| GW_rep_c1248 | 2036 | 21 | 36 | 0.777607578663552 | Up | 0.608662 |
| GW_c48003 | 513 | 0 | 4 | 11.9657842846621 | Up | 0.150248 |
| GW_rep_c59844 | 795 | 1 | 4 | 2 | Up | 0.454306 |
| GW_rep_c13932 | 581 | 4 | 5 | 0.321928094887362 | Up | 0.754532 |
| GW_rep_c77809 | 655 | 0 | 3 | 11.5507467853832 | Up | 0.252146 |
| GW_rep_c9149 | 547 | 2 | 8 | 2 | Up | 0.227124 |
| GW_c33530 | 1109 | 2 | 4 | 1 | Up | 0.821984 |
| GW_rep_c83825 | 412 | 0 | 2 | 10.9657842846621 | Up | 0.423152 |
| GW_rep_c1397 | 1418 | 19 | 55 | 1.53343220008107 | Up | 0.00918174 |
| GW_rep_c74528 | 630 | 0 | 2 | 10.9657842846621 | Up | 0.423152 |
| GW_rep_c1412 | 1696 | 13 | 19 | 0.547487795302493 | Up | 0.94464 |
| GW_rep_c6753 | 777 | 6 | 7 | 0.222392421336448 | Up | 0.637966 |
| GW_c44242 | 1345 | 0 | 4 | 11.9657842846621 | Up | 0.150248 |
| GW_c24605 | 1602 | 1 | 7 | 2.8073549220576 | Up | 0.135117 |
| GW_rep_c68861 | 375 | 1 | 2 | 1 | Up | 0.936496 |
| GW_rep_c532 | 1108 | 2 | 30 | 3.90689059560852 | Up | 9.5776e-05 |
| GW_rep_c101396 | 645 | 3 | 0 | -11.5507467853832 | Down | 0.0533444 |
| GW_rep_c86308 | 510 | 2 | 1 | -1 | Down | 0.367898 |
| GW_rep_c53155 | 461 | 0 | 2 | 10.9657842846621 | Up | 0.423152 |
| GW_rep_c18862 | 1127 | 5 | 6 | 0.263034405833794 | Up | 0.69153 |
| GW_rep_c7398 | 1474 | 9 | 23 | 1.3536369546147 | Up | 0.1693558 |
| GW_rep_c65506 | 727 | 7 | 8 | 0.192645077942396 | Up | 0.591438 |
| GW_rep_c9470 | 487 | 1 | 3 | 1.58496250072116 | Up | 0.660144 |
| GW_c6008 | 1127 | 3 | 5 | 0.736965594166206 | Up | 0.944606 |
| GW_rep_c36948 | 1149 | 4 | 11 | 1.4594316186373 | Up | 0.31671 |
| GW_rep_c86536 | 1024 | 0 | 4 | 11.9657842846621 | Up | 0.150248 |
| GW_rep_c3556 | 1114 | 11 | 34 | 1.62803122261304 | Up | 0.0290622 |
| GW_rep_c66740 | 1172 | 3 | 4 | 0.415037499278844 | Up | 0.830978 |
| GW_rep_c57130 | 914 | 0 | 5 | 12.2877123795495 | Up | 0.0895292 |
| GW_rep_c57893 | 1207 | 5 | 21 | 2.0703893278914 | Up | 0.0278574 |
| GW_rep_c62088 | 1106 | 3 | 7 | 1.22239242133645 | Up | 0.573268 |
| GW_rep_c1092 | 1280 | 50 | 68 | 0.443606651475615 | Up | 0.64941 |
| GW_c15584 | 1159 | 1 | 6 | 2.58496250072116 | Up | 0.204798 |
| GW_c6928 | 1504 | 1 | 10 | 3.32192809488736 | Up | 0.0372072 |
| GW_rep_c54716 | 1510 | 3 | 10 | 1.73696559416621 | Up | 0.236114 |
| GW_rep_c20652 | 715 | 1 | 4 | 2 | Up | 0.454306 |
| GW_c9898 | 944 | 5 | 3 | -0.736965594166206 | Down | 0.208034 |
| GW_rep_c106055 | 383 | 2 | 0 | -10.9657842846621 | Down | 0.1320002 |
| GW_rep_c316 | 1232 | 14 | 29 | 1.05062607306997 | Up | 0.313294 |
| GW_rep_c66483 | 478 | 0 | 2 | 10.9657842846621 | Up | 0.423152 |
| GW_c23219 | 856 | 1 | 3 | 1.58496250072116 | Up | 0.660144 |
| GW_rep_c30852 | 843 | 2 | 3 | 0.584962500721156 | Up | 0.92839 |
| GW_rep_c7381 | 587 | 0 | 5 | 12.2877123795495 | Up | 0.0895292 |
| GW_rep_c18862 | 1127 | 5 | 6 | 0.263034405833794 | Up | 0.69153 |
| GW_rep_c7398 | 1474 | 9 | 23 | 1.3536369546147 | Up | 0.1693558 |
| GW_rep_c1386 | 3089 | 12 | 36 | 1.58496250072116 | Up | 0.0295838 |
